# Functional role of Ash2l in oxLDL induced endothelial dysfunction and atherosclerosis

**DOI:** 10.1007/s00018-024-05130-5

**Published:** 2024-01-27

**Authors:** Zhenghua Su, Jinghuan Wang, Chenxi Xiao, Wen Zhong, Jiayao Liu, Xinhua Liu, Yi Zhun Zhu

**Affiliations:** 1 School of Pharmacy, Human Phenome Institute, Fudan University, Shanghai, 201203 China; 2 State Key Laboratory of Quality Research in Chinese Medicine, School of Pharmacy and 1st affiliate hospital, Macau University of Science and Technology, Macau, China; 3 School of Pharmacy, Macau University of Science and Technology Taipa, Macau, China; 4 Pharmacophenomics Laboratory, Human Phenome Institute, Fudan University, 825, Zhangheng Road, Pudong New District, Shanghai, China

**Keywords:** Ash2l, Endothelial cells, Atherosclerosis, PPARγ, Scavenger receptors, NF-κB

## Abstract

Endothelial injury and dysfunction in the artery wall fuel the process of atherosclerosis. As a key epigenetic regulator, Ash2l (Absent, small, or homeotic-Like 2) is involved in regulating vascular injury and its complications. However, the role of Ash2l in atherosclerosis has not yet been fully elucidated. Here, we found increased Ash2l expression in high-cholesterol diet-fed *ApoE*^*−/−*^ mice and oxidized LDL (oxLDL) treated endothelial cells (ECs). Furthermore, Ash2l promoted the scavenger receptors transcription by catalyzing histone H3 lysine 4 (H3K4) trimethylation at the promoter region of transcription factor peroxisome proliferator-activated receptor-γ (PPARγ) and triggered the activation of the pro-inflammatory nuclear factor-kappa B (NF-κB) by enhancing interaction between CD36 and toll-like receptor 4 (TLR4). Meanwhile, enhanced expression of scavenger receptors drove more oxLDL uptake by ECs. In vivo studies revealed that ECs-specific Ash2l knockdown reduced atherosclerotic lesion formation and promoted fibrous cap stability in the aorta of *ApoE*^*−/−*^ mice, which was partly associated with a reduced endothelial activation by suppressing scavenger receptors and the uptake of lipids by ECs. Collectively, our findings identify Ash2l as a novel regulator that mediates endothelial injury and atherosclerosis. Targeting Ash2l may provide valuable insights for developing novel therapeutic candidates for atherosclerosis.

**Graphical Abstract:**

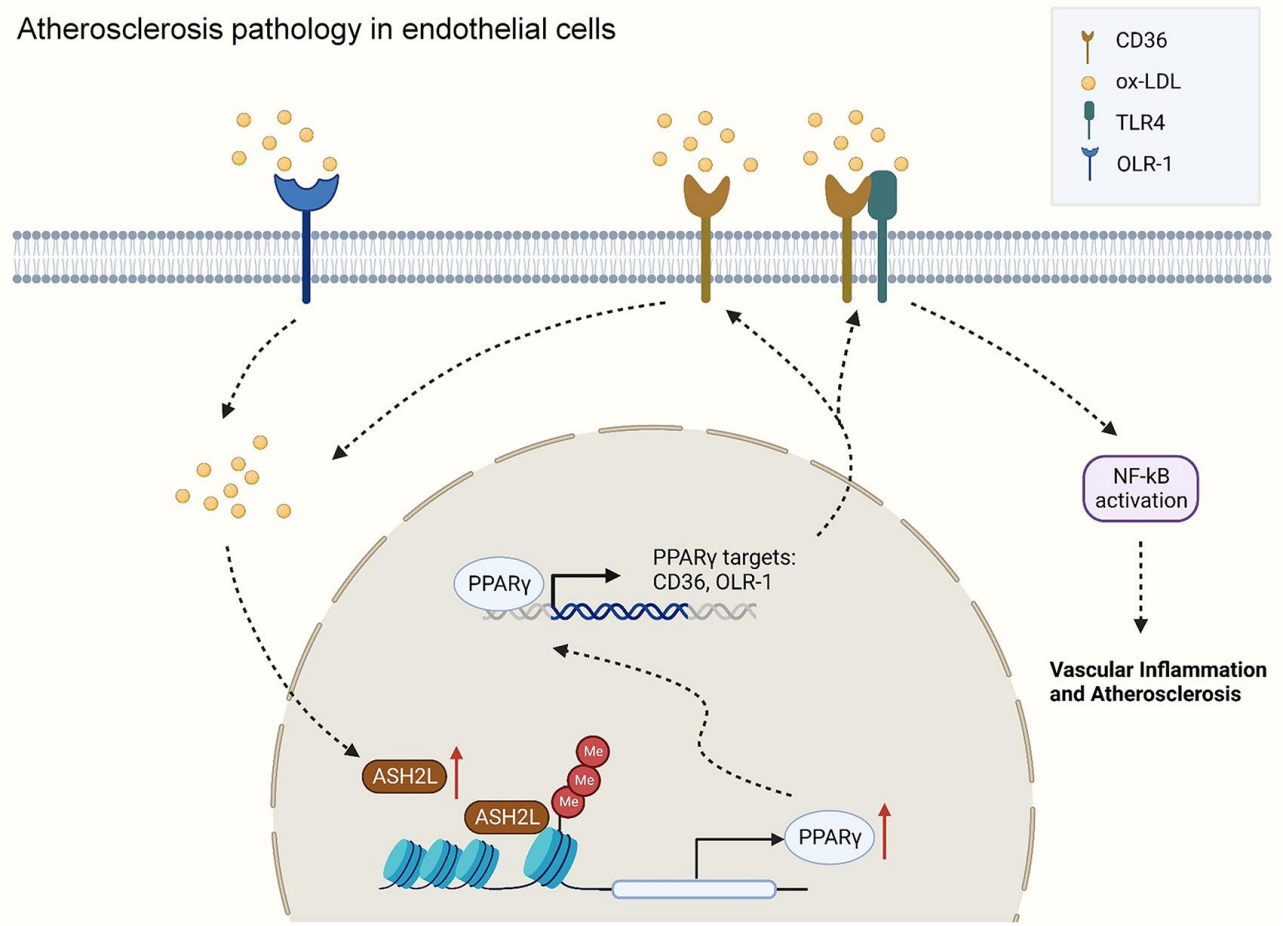

**Supplementary Information:**

The online version contains supplementary material available at 10.1007/s00018-024-05130-5.

## Introduction

Atherosclerosis has been recognized as a multifactorial inflammatory disease characterized by progressive accumulation of atherosclerotic plaque in the arterial wall [1–3]. Cells involved in the atherosclerotic process include vascular (endothelial and smooth muscle) cells, monocytes/macrophages, lymphocytes, dendritic cells, and mast cells [4]. Endothelial cells (ECs) are the first barrier of the vascular system against body damage. The destruction of endothelial homeostasis is the early stage of atherosclerosis and eventually exacerbates vascular injury and the development of vulnerable plaques, causing focal arterial occlusion and other clinical events [5]. Therefore, strategies aimed to maintain the normal barrier function of ECs under high-fat conditions can slow the lesion progression, which may be a potential way for prevention and early treatment of atherosclerosis in combination with standard lipid-lowering therapies.

It has been well established that epigenetic modification plays a crucial role in the regulation of cardiovascular diseases, including atherosclerosis [6]. Mixed-lineage leukemia (MLL) complexes are homologs of yeast COMPASS, responsible for histone H3 lysine 4 (H3K4) trimethylation and associated with actived transcription [7–9]. The methyltransferase activity of the complex depends on its core subunits WDR5, Ash2l (Absent, small, or homeotic-Like 2), RBP5, and DPY30 (WRAD) [9]. Recent investigations indicate that Ash2l mediates embryogenesis and the carcinogenic process. In addition, evidence has suggested that H3K4 methylation is essential for expressing endothelial nitric-oxide synthase (eNOS), a critical molecule for ECs function [10, 11]. However, the effect of Ash2l on endothelial injury and atherosclerosis is still unknown.

Oxidized lipoprotein (oxLDL) as a major autoantigen promotes plaque formation and destabilization, is recognized by several receptors, including scavenger receptor class A (SR-A), CD36, Lectin-like oxidized LDL receptor-1 (OLR-1), CD68, and toll-like receptors (TLRs) [12]. As the primary receptor, OLR-1 facilitates oxLDL uptake by ECs [13, 14]. Endothelial dysfunction is one of the earliest manifestations of atherosclerosis and oxLDL has been recognized as a significant cause of endothelial dysfunction in atherogenesis [5, 15]. OxLDL affects endothelium-dependent vascular tone through loss of nitric oxide (NO) bioavailability and mediates several biological effects via scavenger receptors, such as CD36 [16] and OLR-1 [17, 18]. Corroboratively, OLR-1 expression is upregulated in human and animal atherosclerotic lesions [19]. Notably, previous studies indicate that the scavenger receptors are transcriptionally regulated by peroxisome proliferator-activated receptor-γ (PPARγ), which further promotes oxLDL uptake by cells [20]. In addition, in the formation of early fatty streaks, the endothelium is activated and expresses chemokines and cytokines, including IL-6, MCP-1, intercellular adhesion molecule (ICAM)-1, and vascular adhesion molecule (VCAM)-1, which lead to immune cell recruitment and infiltration into the sub-endothelium, exacerbating inflammatory response and the pathological process of atherosclerosis [21]. Given ECs function in atherosclerosis, identifying the underlying mechanisms that control endothelial activation and dysfunction may lead to the discovery of a treatment strategy.

In this study, we identified that Ash2l as an important regulator in the process of atherosclerosis, enhanced the expression of scavenger receptors through the activation of PPARγ. We found that upregulated scavenger receptors drove excessive oxLDL uptake by ECs and triggered activation of the pro-inflammatory nuclear factor-kappa B (NF-κB) by enhancing the interaction with TLR4. ECs-specific Ash2l knockdown reduced atherosclerotic lesion formation and promoted fibrous cap stability in the aorta of *ApoE*^−/−^ mice. Ash2l, therefore, represents a potential intervention for atherosclerosis-mediated endothelial dysfunction.

## Materials and methods

The data supporting this study’s findings are available from the corresponding author upon reasonable request. An expanded Materials and methods section can be found in the Supplemental materials and methods.

### Statistical analysis

GraphPad Prism 8.0 was used to analyze the data which were expressed as mean ± SD. Differences of means were analyzed using one-way ANOVA to compare one variable in multiple groups, and when comparing two groups using unpaired Students t-test. *P* < 0.05 was regarded as statistically significant.

## Results

### Ash2l was abundantly expressed in oxLDL-induced ECs and within atherosclerotic plaques in *ApoE*^−/−^ mice

**Fig. 1:**
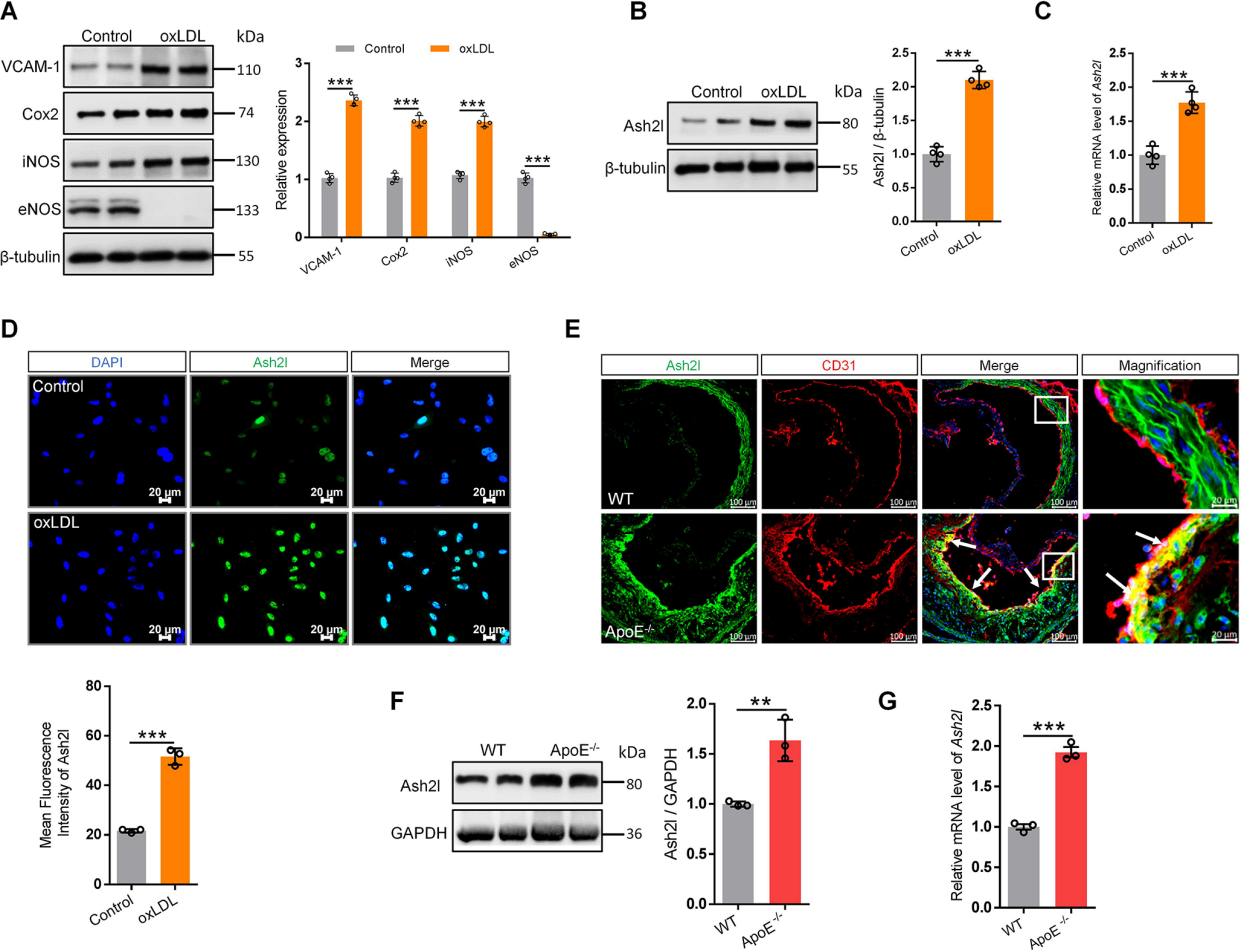
Ash2l is abundantly expressed in oxLDL-induced ECs and within atherosclerotic plaques in *ApoE*^−/−^ mice. **A–D** OxLDL (50 µg/mL, 24 h)-induced rat artery endothelial cells (RAECs) were used as the in vitro atherosclerosis model. **A** Immunoblots analysis and quantification for endothelial inflammation (VCAM-1, Cox2, iNOS) and dysfunction (eNOS) associated markers, *n* = 4. The protein expression **B** and mRNA level **C** of *Ash2l* were detected by immunoblots and qPCR, respectively, *n* = 4. Quantification of Ash2l expression (**B** Right panel) was presented relative to that of β-tubulin (loading control). **D** Immunofluorescence detection of Ash2l expression and location in oxLDL treated or untreated RAECs; scale bars, 20 μm. Bottom panel: quantification for the MFI of Ash2l in each group, *n* = 3. **E–G** The in vivo atherosclerosis model was established by feeding *ApoE*^−/−^ mice with high-cholesterol diet for 12 weeks and the aortic arteries were harvested for the subsequent studies, WT mice feeding with normal chow diet as control. **E** Immunofluorescence staining of Ash2l (green) and CD31 (red) and their co-localization (yellow merge; see white arrows) in aortic sinus of mice; scale bars, 100 μm or 20 μm. Immunoblot analysis **F** and qPCR analysis **G** for Ash2l in the aorta of mice, *n* = 3. Quantification of Ash2l expression in **F** (right panel) was presented relative to that of GAPDH (loading control). Data were presented as the mean ± S.D, *P* values were calculated by two-tailed Student’s t-test, ^**^p < 0.01, ^***^p < 0.001

The pathogenesis of atherosclerosis was complicated, and vascular ECs injury was the early step in the initiation of atherosclerosis. To investigate whether Ash2l was associated with endothelial injury, we treated primary cultured rat artery endothelial cells (RAECs) with oxLDL, a potent inflammatory agent which can induce endothelial inflammation and further lead to endothelial dysfunction. We found that oxLDL dramatically induced the expression of inflammatory mediators such as iNOS, Cox2, VCAM-1 and inhibited a protective molecular endothelial NO synthase (eNOS) (Fig. 1A). Surprisingly, we found that oxLDL treatment could promote Ash2l expression (Fig. 1B) and this was confirmed by RT-qPCR (Fig. 1C). Immunofluorescence staining further explained that oxLDL elevated the expression of Ash2l, especially Ash2l was mainly localized in the nucleus of ECs and may participate in the modification of histones (Fig. 1D). The above data indicated that Ash2l may be involved in oxLDL-induced endothelial injury and dysfunction.

Considering endothelial injury and dysfunction were typical features of early atherosclerotic lesions, we detected Ash2l expression changes on the ECs of aortic sinus plaques from high-cholesterol diet-fed *ApoE*^*−/−*^ mice, an animal model for atherosclerosis. Cross-sections of the aortic root were double stained with antibodies against Ash2l and CD31 (a marker for ECs). The expression of Ash2l was observed on ECs labeled by CD31 and markedly increased in *ApoE*^−/−^ mice compared to WT mice (Fig. 1E). We also lysed the aorta and performed a western blot analysis. Consistent with our immunofluorescence staining results, Ash2l was significantly upregulated in high-cholesterol diet-fed *ApoE*^−/−^ mice compared to WT mice (Fig. 1F). Furthermore, *Ash2l* mRNA level was also significantly increased (Fig. 1G). These data indicated that increased Ash2l levels in the atherosclerotic plaques induced by a high-cholesterol diet may participate in endothelial injury and dysfunction.

### ECs-specific Ash2l knockdown alleviated atherosclerosis

**Fig. 2:**
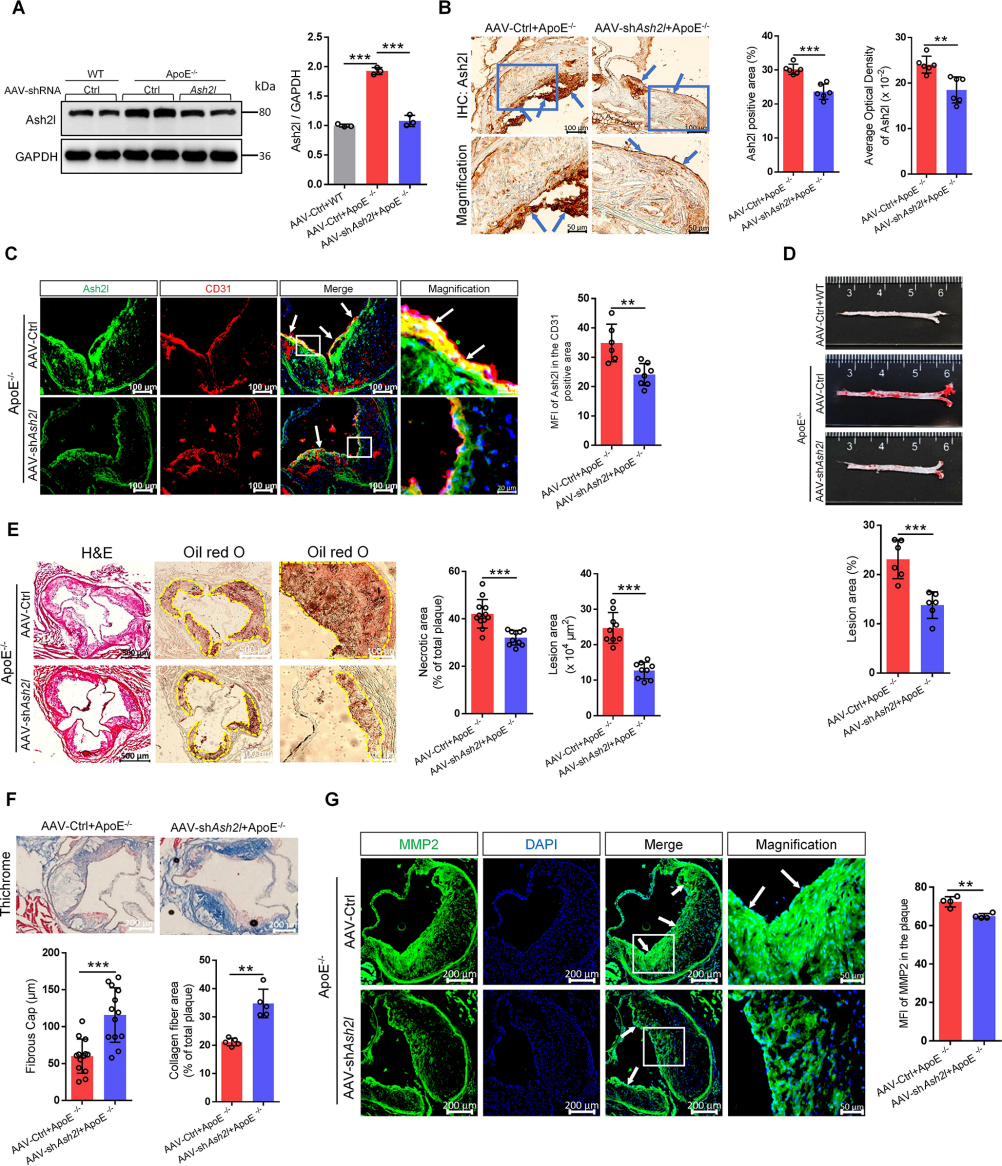
ECs-specific Ash2l knockdown alleviates atherosclerosis. Male *ApoE*^−/−^ mice were injected via caudal vein with ECs-specific AAV-shRNA *Ash2l* or AAV-Ctrl on the first week of high-cholesterol diet and raised up for another 11 weeks, AAV-Ctrl was injected to male C57BL/6J (WT) mice feeding with normal chow diet for 12 weeks as control. **A** Immunoblots and the statistical analysis for Ash2l in the aorta from each group, *n* = 3. **B** Immunohistochemical staining for Ash2l in cross-sections of the aortic roots from each group (see blue arrows); scale bars, 100 μm or 50 μm. The right panel indicated the quantification for Ash2l positive area or average optical density (AOD) of Ash2l, *n* = 6. **C** Ash2l expression (green) was detected by immunofluorescence staining and located with CD31 (red) in the atherosclerotic lesions of each group (yellow merge; see white arrows); scale bars, 100 μm or 20 μm. Right panel: quantification for MFI of Ash2l in the CD31 positive area, *n* = 6–8. **D** Representative images of atherosclerosis in the aorta en-face of each group. The aorta was stained with oil red O. Quantification of lesion area of aortic was shown in the figure bottom, *n* = 6. **E** Representative sections of H&E staining or oil red O staining of lesions in aortic sinus from each group, and the yellow dashed lines indicate the lesion borders; scale bars, 500 μm or 100 μm. Quantification for the lesion area of aortic sinus or quantification for the proportion of necrotic area in total plaques was shown in the figure right, *n* = 9–12. **F** Representative pictures of Masson Trichrome staining showing the collagen (blue) content in cross-sections of aortic sinus from each group; scale bars, 200 μm. Bottom panel: quantification for the fibrous cap thickness or the proportion of collagen fiber area in total plaques, *n* = 5–13. **G** Representative images of Immunofluorescence staining for MMP2 in atherosclerotic lesions from each group (see white arrows); scale bars, 200 μm or 50 μm. Right panel: quantification for MFI of MMP2 in the plaque, *n* = 4. Data were presented as the mean ± S.D, *P* values were calculated by One-way ANOVA test or two-tailed Student’s t-test, ^**^p < 0.01, ^***^p < 0.001

To study the effect of Ash2l ablation on the development of atherosclerosis, Ash2l in ECs was knocked down using ECs-specific *Ash2l* shRNA adeno-associated virus (AAV-sh*Ash2l*) to infect *ApoE*^−/−^ mice, and AAV-shRNA Control (AAV-Ctrl) was injected as control. Compared to WT mice, *ApoE*^−/−^ mice fed a high cholesterol diet for 12 weeks displayed significantly upregulation of Ash2l, whereas that was blocked in AAV-shRNA *Ash2l* treated *ApoE*^−/−^ mice (Fig. 2A), above results were further confirmed by immunohistochemical staining. As shown in Fig. 2B, Ash2l was abundantly expressed in the endothelial layer and near the endothelial layer compared to the other regions of aortic plaques and statistical analysis showed the *ApoE*^−/−^ mice treated with AAV-shRNA *Ash2l* displayed less positive area or average optical density (AOD) of Ash2l than that treated with AAV-Ctrl. Consistently, compared to AAV-Ctrl-injected mice, the mean fluorescent intensity (MFI) of Ash2l in CD31 positive area was weaker in atherosclerotic lesions of AAV-shRNA *Ash2l*-injected mice, as identified by dual immunofluorescence staining of Ash2l and CD31 (Fig. 2C). Vascular Smooth Muscle Cells (VSMCs) and monocytes/macrophages have been also proved to be involved in the process of atherosclerosis, but our immunofluorescence staining results did not show that AAV-shRNA *Ash2l* had an effect on the expression of Ash2l in VSMCs and monocytes/macrophages, which indicated that AAV-shRNA *Ash2l* was targeted at ECs (Fig. S1A and B).

Because lipid metabolism contributed significantly to atherosclerosis, we next examined the effects of Ash2l on multiple metabolic parameters in mice fed with normal chow or high cholesterol diet for 12 weeks. We did not detect obvious differences between the *ApoE*^−/−^ mice injected with AAV-shRNA *Ash2l* and that injected with AAV-Ctrl in serum triglyceride (TG), total cholesterol (TC), low-density lipoprotein cholesterol (LDL-C), and high-density lipoprotein cholesterol (HDL-C) levels (Fig. S2). These data suggested decreased atherosclerotic lesion formation by Ash2l deletion occurred independent of the plasma lipid profiles.

Despite similar lipid profiles, Oil red O staining to the aorta en-face and cross-sections of the aortic root revealed that the *ApoE*^−/−^ mice treated with AAV-shRNA *Ash2l* had an atherosclerotic lesion area smaller than that treated with AAV-Ctrl (Fig. 2D and E). Plaque morphology analysis indicated that AAV-shRNA *Ash2l* treated *ApoE*^−/−^ mice contained smaller necrotic cores than AAV-Ctrl treated *ApoE*^−/−^ mice (Fig. 2E). Histological analysis revealed that the *ApoE*^*−/−*^ mice treated with AAV-shRNA *Ash2l* showed a higher collagen deposition and a thicker fiber cap than those treated with AAV-Ctrl after Masson Trichrome staining (Fig. 2F). There was evidence that the expression and activity of MMPs were related to the stability of atherosclerotic plaque. Enhanced MMP expression and activity may lead to plaque rupture by enhancing collagen breakdown, so cross-sections of aortic root were stained with MMP2 antibody, and the result indicated that compared to *ApoE*^*−/−*^ mice treated with AAV-Ctrl, the MFI of MMP2 in *ApoE*^*−/−*^ mice treated with AAV-shRNA *Ash2l* was significantly decreased, and the positive area of MMP2 was also significantly reduced (Fig. 2G). The above results indicated the relative importance of endothelial Ash2l in atherosclerotic plaque formation and risk of plaque rupture.

### ECs-specific Ash2l knockdown inhibited inflammatory mediators production and scavenger receptors expression in the aorta

**Fig. 3:**
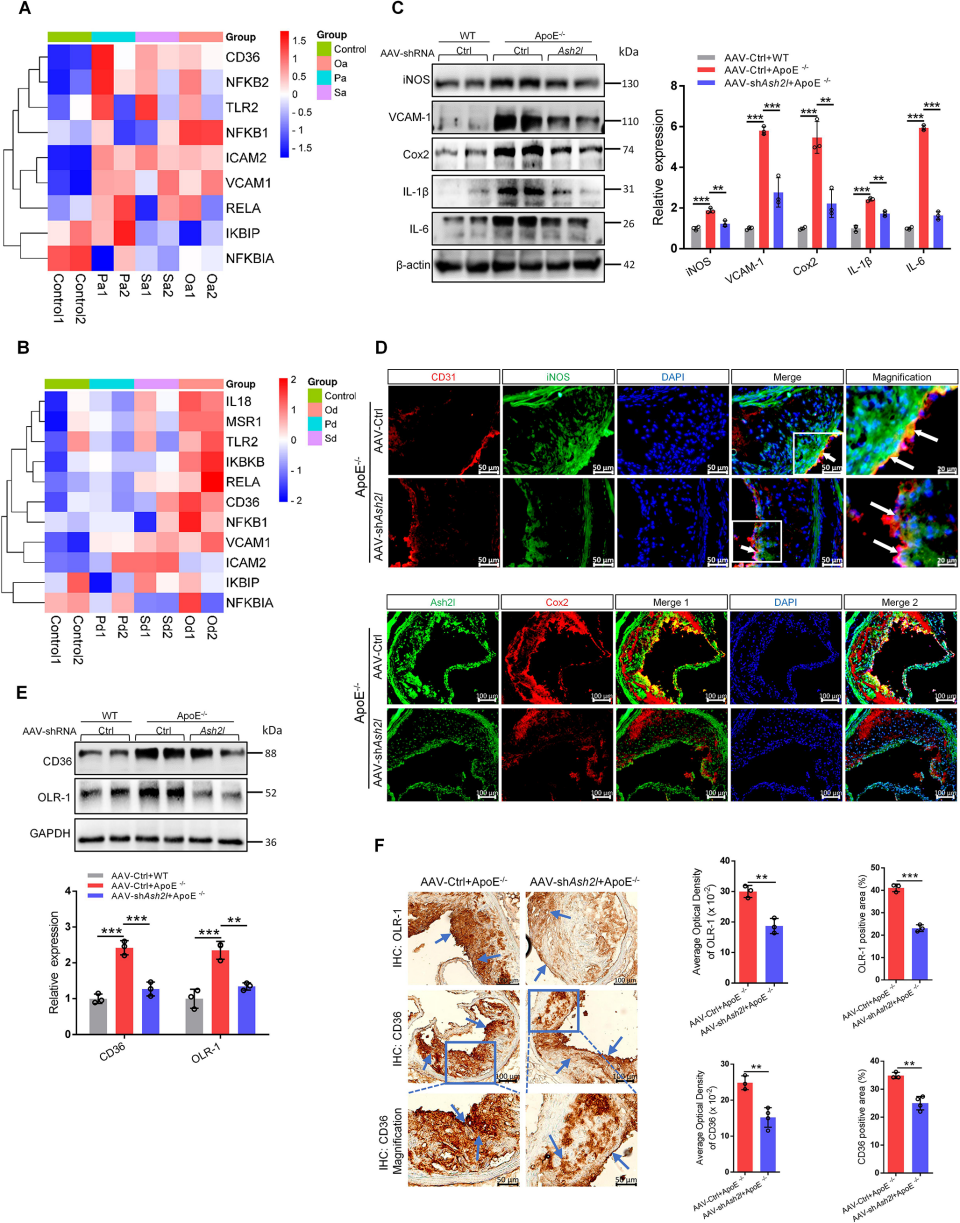
ECs-specific Ash2l knockdown inhibits inflammatory mediators production and scavenger receptors expression in vivo. **A**, **B** Heat map of the differentially expressed genes among pristine, stenosis, and occlusion arteries from patients with atherosclerosis (Pa/Sa/Oa) (**A**) or atherosclerosis and diabetes (Pd/Sd/Od) (**B**). **C**-**F***ApoE*^−/−^ mice were injected via caudal vein with endothelium-specific AAV-shRNA *Ash2l* or AAV-Ctrl on the first week of high-cholesterol diet and raised up for another 11 weeks, AAV-Ctrl was injected to male C57BL/6J (WT) mice feeding with normal chow diet for 12 weeks as control. **C** The expression of inflammatory mediators (iNOS, VCAM-1, Cox2, IL-1β, IL-6) in the aorta isolated from AAV-shRNA *Ash2l* and AAV-Ctrl treated *ApoE*^−/−^ mice or AAV-Ctrl treated WT mice was detected by western blot. The statistical analysis of relative protein expression was shown in the figure right, *n* = 3. **D** Immunofluorescence staining for CD31 (red)/iNOS (green) or Ash2l (green) /Cox2 (red) in cross-sections of the aortic roots from AAV-shRNA *Ash2l* and AAV-Ctrl treated *ApoE*^−/−^ mice; scale bars, 100 μm, 50 μm or 20 μm. **E** Immunoblots analysis for scavenger receptors (CD36, OLR-1) in the aorta from each group. The statistical analysis of relative protein expression was shown in the figure bottom, *n* = 3. **F** Immunohistochemical staining for scavenger receptors (CD36, OLR-1) in atherosclerotic lesions was compared between AAV-shRNA *Ash2l* and AAV-Ctrl treated *ApoE*^−/−^ mice (see blue arrows); scale bars, 100 μm or 50 μm. The right panel indicated the quantification for positive area or AOD of OLR-1 and CD36, *n* = 3–4. Data were presented as the mean ± S.D, *P* values were calculated by One-way ANOVA test or two-tailed Student’s t-test, ^**^p < 0.01, ^***^p < 0.001

Endothelial pro-inflammatory activation was a critical step in the initiation and progression of atherosclerosis. Compared with the control group, we found the differential expression of inflammatory genes, NF-κB signaling molecules and scavenger receptors in pristine (not yet stenosis or occlusion), stenosis, and occlusion arteries of atherosclerosis patients (Pa/Sa/Oa) or atherosclerosis with diabetes patients (Pd/Sd/Od) **(**Fig. 3A and B**)**. We further determined the effect of ECs-specific Ash2l knockdown on inflammatory mediators. Upregulated inflammatory mediators (Cox2, iNOS, IL-6, IL-1β, VCAM-1) were strongly reduced in the aortas of *ApoE*^*−/−*^ mice treated with AAV-shRNA *Ash2l* when compared to AAV-Ctrl treatment (Fig. 3C). Immunostaining for aortas sinus sections also confirmed the reduced expression of iNOS, Cox2 and ICAM-1 in the lesions of *ApoE*^−/−^ mice treated with AAV-shRNA *Ash2l*, including in ECs (Fig. 3D and S3A).

As expected, the scavenger receptors (CD36 and OLR-1) protein levels also showed a marked decrease in the aortas of *ApoE*^−/−^ mice treated with AAV-shRNA *Ash2l* when compared to AAV-Ctrl treatment (Fig. 3E). The aortic plaques staining results indicated that CD36 or OLR-1 was more expressed in the endothelial layer and near endothelial layer compared to internal plaques. And further analysis of stained plaques showed a higher percent in positive area, MFI and AOD of scavenger receptors in AAV-shRNA *Ash2l* group, whereas the percentage of them was lower in AAV-Ctrl group (Fig. 3F and S3B).

Notably, we also observed the scavenger receptors expression in the plaques center, especially CD36, which was mainly contributed by monocytes/macrophages or foam cells (Fig. 3F and S3B). Changing the characteristics of ECs may affect the extravasation of monocytes/macrophages into the tissue, but the immunohistochemical staining result of CD68 indicated that ECs-specific Ash2l knockdown seemed to have little effect on the numbers and the influx of monocytes/macrophages (Fig. S4).

The above results illustrated that Ash2l mediated inflammatory mediators production and scavenger receptors expression in ECs, while blockade of Ash2l served as endothelial protection and anti-inflammatory effects that may also be anti-atherosclerosis.

### Knockdown of Ash2l decreased scavenger receptors expression and DiI-oxLDL uptake by ECs, and protected against endothelial injury and dysfunction

**Fig. 4:**
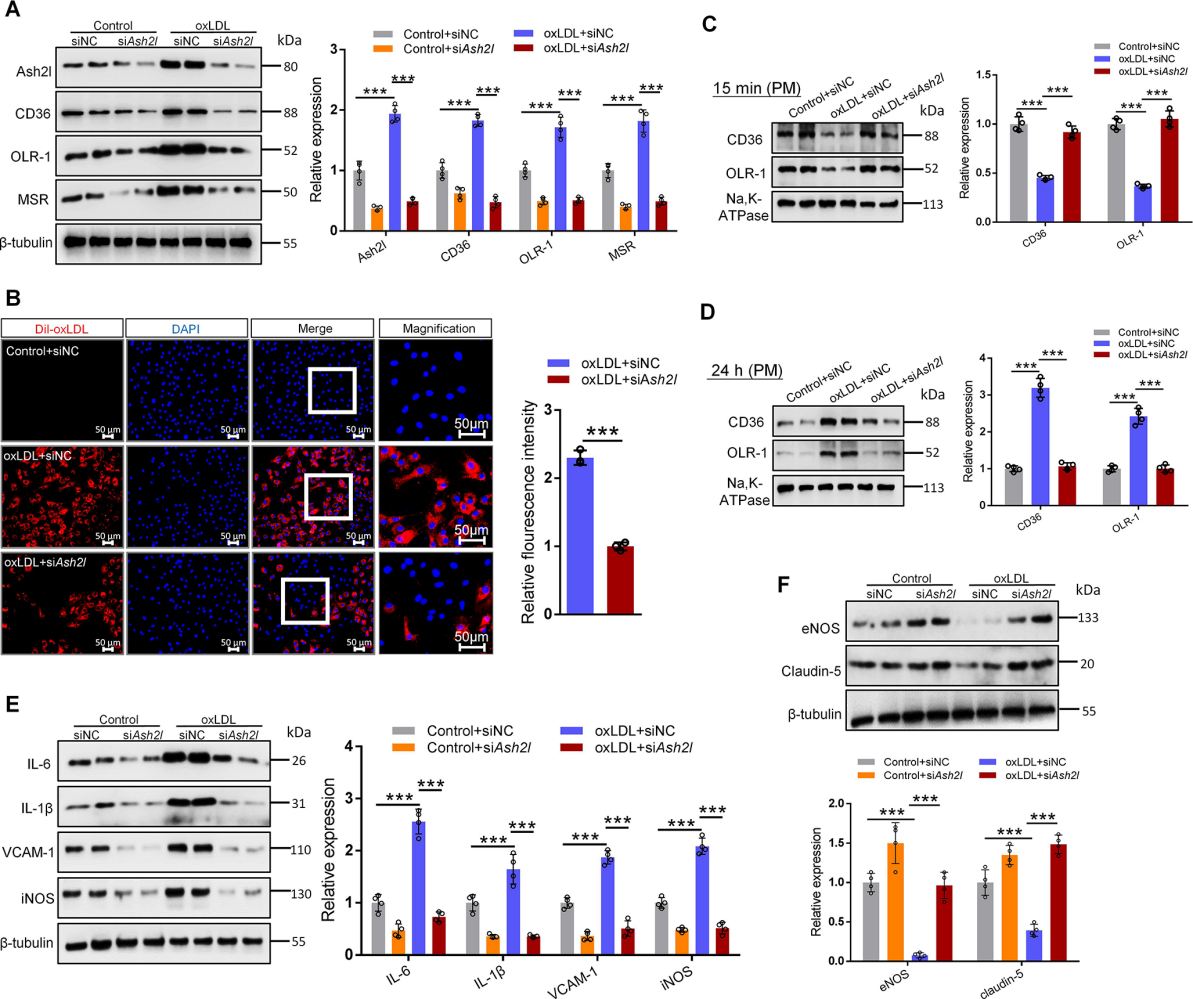
Knockdown of Ash2l decreases scavenger receptors expression and DiI-oxLDL uptake by ECs, and protects against endothelial injury and dysfunction. Ash2l was knocked down using siRNA *Ash2l* in oxLDL (50 µg/mL, 24 h) induced RAECs, siRNA control (siNC) was used as the negative control. **A** Immunoblots and quantification of Ash2l and scavenger receptors (CD36, OLR-1, MSR), β-tubulin was used as a loading control, *n* = 4. **B** RAECs were incubated with Dil-oxLDL (50 µg/mL) for 4 h. Total uptake of Dil-oxLDL was detected by fluorescence microscope; scale bars, 50 μm. Quantification for Dil-oxLDL uptake was shown in the figure right, *n* = 3. **C**, **D** Immunoblots analysis for membrane CD36 and OLR-1 in RAECs treated with oxLDL for 15 min (**C**) or 24 h (**D**), Na, K-ATPase served as a loading control. Quantification of CD36 and OLR-1 protein level relative to Na, K-ATPase was shown in the figure right, *n* = 4. **E** Immunoblots analysis and quantification for inflammatory mediators (iNOS, VCAM-1, IL-1β, IL-6) in oxLDL (50 µg/mL, 24 h) induced RAECs, β-tubulin served as a loading control, *n* = 4. **F** Immunoblots analysis and quantification for eNOS and Claudin-5 in oxLDL treated RAECs, β-tubulin was used as a loading control, *n* = 4. Data were presented as the mean ± S.D, *P* values were calculated by two-tailed Student’s t-test **B** or One-way ANOVA test (**A**, **C**–**F**), ^***^p < 0.001

Since our above results demonstrated that ECs-specific Ash2l deficiency inhibited atherosclerotic plaque progression and scavenger receptors expression in *ApoE*^−/−^ mice, we asked whether Ash2l had the same effect in oxLDL-induced ECs. Therefore, we used siRNA targeting *Ash2l* to silence its expression in ECs. In agreement with in vivo results, compared to oxLDL challenge, Ash2l knockdown showed a remarkable reduction of CD36, OLR-1, or MSR expression in both RAECs and HUVECs (Fig. 4A and S5A). It has been reported that ECs could transport oxLDL to subintima through transcytosis, leading to lipid accumulation and plaque formation. Because the internalization of scavenger receptors was a crucial step for transcytosis in ECs, we hypothesized that Ash2l might facilitate scavenger receptors internalization after stimulation with oxLDL. We next treated ECs with Dil-labeled oxLDL to trace oxLDL uptake. As shown in Fig. 4B, the oxLDL uptake by ECs was significantly impaired through Ash2l knockdown. Consistent with this finding, in basal conditions, CD36 and OLR-1 were predominantly located on the plasma membrane, whereas during oxLDL exposure, CD36 and OLR-1 were internalized to intracellular vesicles, and the internalization of membrane CD36 and OLR-1 was significantly inhibited by transfecting with *Ash2l* siRNA (Fig. 4C). These data supported the fact that Ash2l regulated scavenger receptors internalization and oxLDL uptake. We also focused on whether Ash2l regulated the expression of scavenger receptors on the membrane, so we extended the incubation time (24 h) of oxLDL on EC and obtained the membrane fraction for western blot experiments. The result indicated oxLDL upregulated the expression of scavenger receptors on the membrane, which was significantly inhibited by Ash2l knockdown (Fig. 4D**).**

Damaged ECs secreted more inflammatory factors and adhesion molecules, leading to vascular injury and atherosclerosis. So, we next performed a Western blot assay to verify whether Ash2l knockdown protected against endothelial injury in response to oxLDL stimulation. As expected, Ash2l knockdown significantly suppressed oxLDL-induced inflammatory mediators, such as iNOS, IL-6, IL-1β and VCAM-1 in both RAECs and HUVECs (Fig. 4E and S5B). Compared with the control group, oxLDL also significantly decreased eNOS and Claudin-5 expression. In contrast, the low levels expression of eNOS and Claudin-5 was reversed by knocking down of Ash2l (Fig. 4F). These findings, consistent with the phenotype in vivo, that Ash2l knockdown significantly improved the endothelial dysfunction and inflammatory response caused by oxLDL in ECs, which was beneficial to the early stage of atherosclerosis formation.

### Ash2l overexpression promoted scavenger receptors expression and oxLDL uptake by ECs, and enhanced ECs inflammatory response

**Fig. 5:**
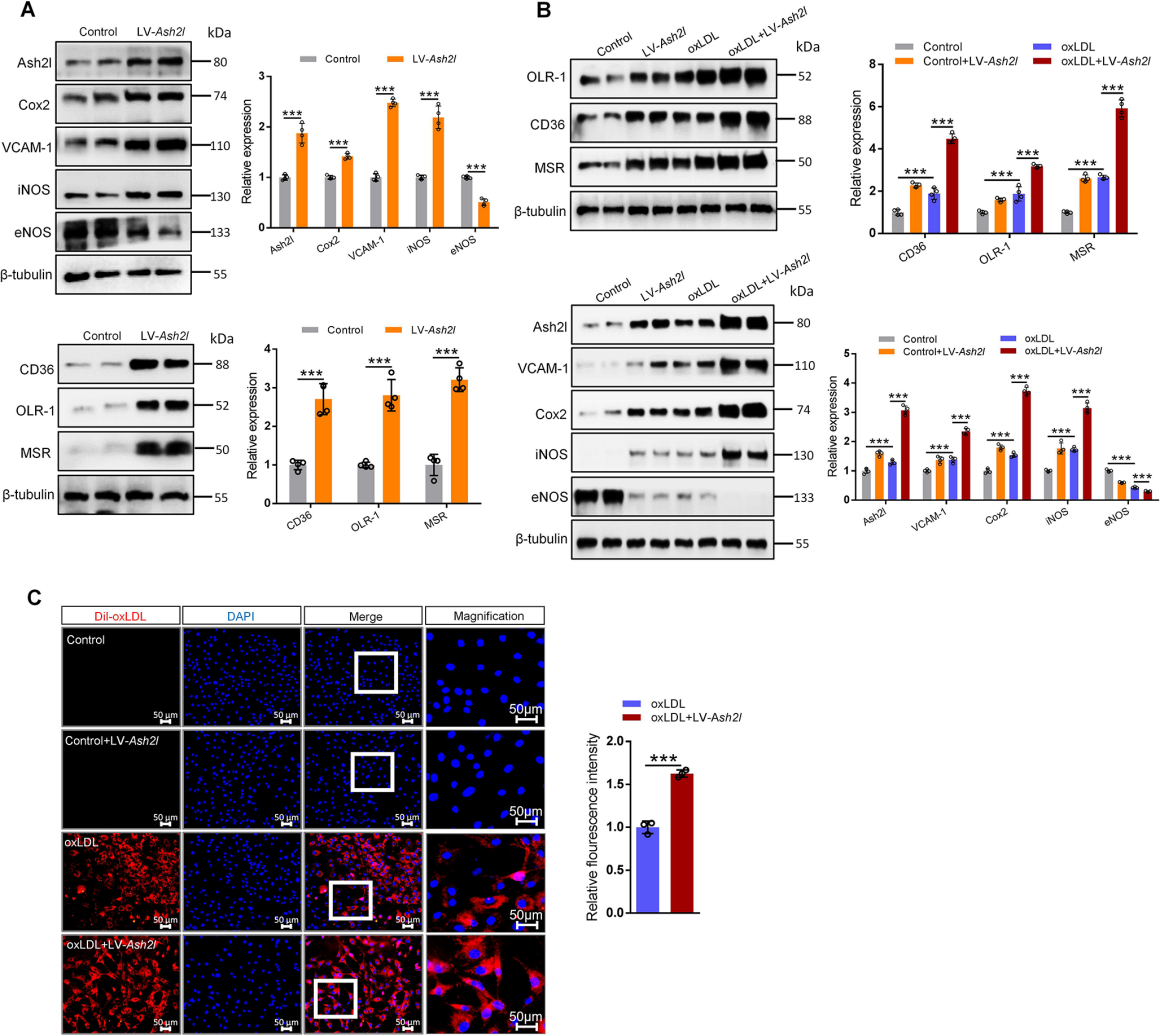
Ash2l overexpression promotes scavenger receptors expression and oxLDL uptake by ECs, and enhances ECs inflammatory response. Ash2l was overexpressed (Ash2l OE) in RAECs by infecting with lentivirus-mediated *Ash2l* cDNA. **A**, **B** Immunoblots analysis and quantification for Ash2l, eNOS, inflammatory mediators (iNOS, VCAM-1, Cox2) and scavenger receptors (CD36, OLR-1, MSR) in Ash2l-overexpressed RAECs treated with or without oxLDL for 24 h. β-tubulin served as a loading control, *n* = 4. **C** RAECs pre-infected with lentivirus-mediated *Ash2l* cDNA were incubated with or without Dil-oxLDL (50 µg/mL) for 4 h. Total uptake of Dil-oxLDL was detected by fluorescence microscope, scale bars; 50 μm. Quantification of the Dil-oxLDL uptake was shown in the figure right, *n* = 3. All data were presented as the mean ± S.D, *P* values were calculated by two-tailed Student’s t-test **A**, **C** or One-way ANOVA test **B**, ^***^p < 0.001

Our above data have shown that Ash2l knockdown improved ECs injury induced by oxLDL. To further demonstrate that Ash2l overexpression enhanced ECs inflammatory response and oxLDL uptake, we infected ECs with lentivirus-mediated *Ash2l* (LV-*Ash2l*) cDNA. Compared to control group, LV-*Ash2l* cDNA efficiently increased Ash2l expression in ECs (Fig. 5A). Notably, the expression of inflammatory mediators (Cox2, iNOS, VCAM-1) and scavenger receptor genes (CD36, OLR-1, MSR) were also increased upon Ash2l overexpression both in ECs treated with or without oxLDL, on contrary, the expression of protective molecular eNOS was inhibited (Fig. 5A and B). We next assessed the effect of LV-*Ash2l* cDNA infection on oxLDL uptake in ECs. Indeed, Ash2l-overexpressed ECs enhanced Dil-oxLDL uptake, pointing to aggravation of atherosclerosis upon Ash2l overexpression (Fig. 5C). Collectively, these data suggested that Ash2l augmented ECs inflammatory mediators production, which may be drived by the enhancement expression of scavenger receptors and the increased uptake of oxLDL.

### Enriched Ash2l at the promoter of PPARγ enhanced the scavenger receptors expression in oxLDL treated ECs

**Fig. 6:**
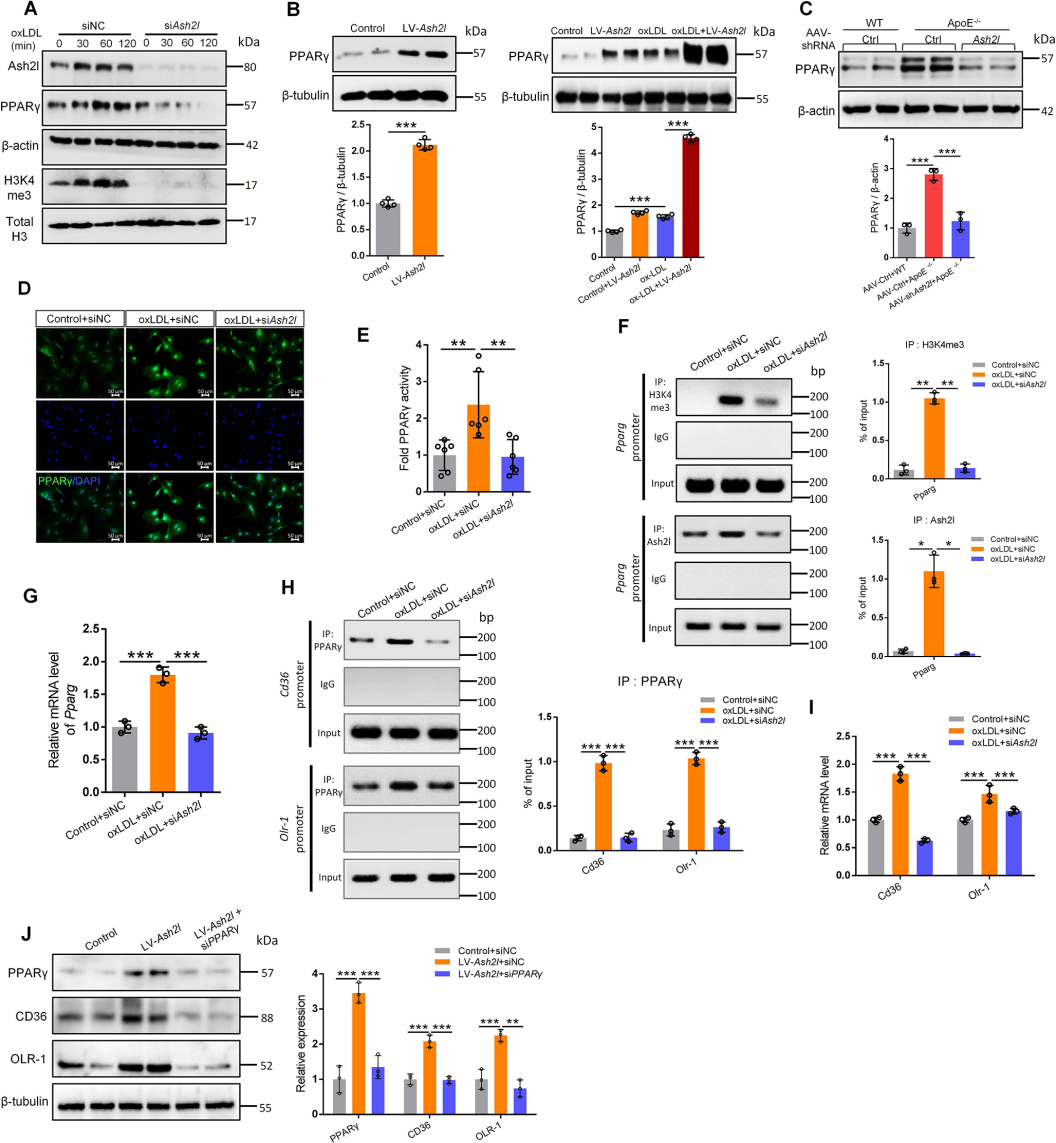
Enriched Ash2l at the promoter of PPARγ enhances the scavenger receptors expression in oxLDL treated ECs. **A** RAECs pre-transfected with siRNA *Ash2l* or siRNA control were incubated with 50 µg/mL oxLDL for indicated time, Ash2l, PPARγ and H3K4me3 protein levels were detected. β-actin was used as a loading control of Ash2l and PPARγ, whereas total H3 served as a loading control of H3K4me3. **B** RAECs pre-infected with lentivirus-mediated *Ash2l* cDNA were incubated with or without oxLDL, immunoblots analysis and quantification for PPARγ was shown in figure, β-tubulin served as a loading control, *n* = 4. **C** Immunoblots and the statistical analysis for PPARγ in the aorta from each group, β-actin served as a loading control, *n* = 3. **D** Immunofluorescence staining for PPARγ (green) in oxLDL induced RAECs pre-transfected with siRNA *Ash2l* or siRNA control; scale bars, 50 μm. **E** PPARγ activity was measured in oxLDL induced RAECs pre-transfected with siRNA *Ash2l* or siRNA control, *n* = 6. **F** ChIP-qPCR assay was performed to determine the H3K4me3 and Ash2l level at the *Pparg* promoter region in oxLDL induced RAECs pre-transfected with siRNA *Ash2l* or siRNA control, the visualization and quantification of the gel for the PCR products were shown in figure, *n* = 3. **G** qPCR analysis for *Pparg* mRNA level in oxLDL induced RAECs pre-transfected with siRNA *Ash2l* or siRNA *control*, *n* = 3. **H** ChIP-qPCR assay was performed to determine the combination of PPARγ and the promoter region of Scavenger receptors (*Cd36, Olr-1*) in oxLDL induced RAECs, the visualization and quantification of the gel for the PCR products were shown in figure *n* = 3. **I** qPCR analysis for *Cd36* and *Olr-1* mRNA level in oxLDL induced RAECs pre-transfected with siRNA *Ash2l* or siRNA control, *n* = 3. **J** RAECs were pre-infected with lentivirus-mediated *Ash2l* cDNA and then transfected with siRNA *PPARγ*, immunoblots analysis was performed to determine protein expression of Ash2l, PPARγ, CD36 and OLR-1, quantification of these protein expression was shown in the figure right, *n* = 3. All data were presented as the mean ± S.D, *P* values were calculated by two-tailed Student’s t-test **(B)** or One-way ANOVA test **(B-C, E-J)**, ^*^p < 0.05, ^**^p < 0.01, ^***^p < 0.001

OxLDL has been proved to be a potent activator of PPARγ and could facilitate PPARγ nuclear translocation and subsequent expression of scavenger receptors. As shown in Fig. 6A, incubation of ECs with oxLDL lead to PPARγ gradually enhancing expression starting from 30 min, and this tendency was reversed significantly in ECs pretreated with *Ash2l* siRNA. Correspondingly, the PPARγ protein level in Ash2l-overexpressed ECs increased to 3.0-fold after oxLDL stimulation (Fig. 6B). In vivo, the expression of PPARγ was greatly reduced in the aorta of *ApoE*^*−/−*^ mice treated with AAV-shRNA *Ash2l* compared to that treated with AAV-Ctrl (Fig. 6C). Furthermore, oxLDL-induced nuclear translocation of PPARγ was observed to be markedly decreased in Ash2l-knockdown ECs by immunostaining (Fig. 6D). To further demonstrate that Ash2l could influence the PPARγ activation, we infected ECs with a construct encoding a PPARγ reporter gene, and we found that oxLDL increased the activity of PPARγ luciferase reporter in ECs but did not affect that in Ash2l knockdown ECs (Fig. 6E**)**.

Given that Ash2l could promote gene expression by mediating and enhancing the H3K4 methylation, we detected the level of H3K4me3 through immunoblot. The results indicated that oxLDL could induce high level of H3K4me3, however, when Ash2l was knocked down, H3K4 trimethylation was gone (Fig. 6A). The identical changes of H3K4me3 and PPARγ under different conditions suggested that Ash2l might regulate PPARγ expression by promoting H3K4 trimethylation, which was further confirmed by the ChIP followed by qPCR (ChIP-qPCR) experiments. The results indicated Ash2l and H3K4me3 were enriched at the promoter region of *Pparg* in ECs exposed to oxLDL **(**Fig. 6F and S5C**)** and promoted *Pparg* transcription (Fig. 6G). While Ash2l knockdown abolished Ash2l from binding to *Pparg* DNA and decreased the mRNA level of *Pparg*, further emphasizing that Ash2l preferentially bound to actively transcribed genes through a H3K4me3 dependent way.

To understand whether Ash2l-mediated PPARγ activation promoted *Cd36* and *Olr-1* transcription, we next performed ChIP-qPCR experiments using an anti-PPARγ antibody. Indeed, ChIP assays using primer spanning 0-500 bp region at the *Cd36* and *Olr-1* gene promoter showed the PPARγ binding to the promoter, which was further augmented by oxLDL **(**Fig. 6H and S5D**)**. Consistently, knocking down of Ash2l inhibited *Cd36* and *Olr-1* transcription **(**Fig. 6I**)**. Collectively, these results strongly suggested that transcription activation of the PPARγ signaling pathway was involved in Ash2l-mediated transcription of scavenger receptors.

To further validate whether the effect of Ash2l on scavenger receptors expression was dependent on PPARγ, we used PPARγ agonist Troglitazone in Ash2l-knocked down ECs. As expected, after PPARγ reactivation, decreased expression of scavenger receptors genes (CD36, OLR-1, MSR) was almost completely recovered, confirming that Ash2l promoted scavenger receptors expression by activating PPARγ (Fig. S6A). Next, we assessed whether high expression of scavenger receptors genes caused by overexpression of Ash2l could be compromised blockade by PPARγ, we performed combat experiments using siRNA *PPARγ* or PPARγ antagonists T0070907 in Ash2l-overexpressed ECs, and found that the high expression of scavenger receptors (CD36, OLR-1, MSR) induced by LV-*Ash2l* was partly blocked by siRNA *PPARγ* or T0070907 (Fig. 6J and S6B). The results also indicated siRNA *PPARγ* treatment inhibited the upregulation of inflammatory mediators (Cox2, iNOS, IL-6, IL-1β, VCAM-1) induced by LV-*Ash2l* (Fig. S6C). Collectively, these results indicated that PPARγ, as a key transcription factor, was regulated by Ash2l and further transcriptionally activated scavenger receptors in ECs, thereby promoting oxLDL uptake.

### Ash2l promoted the interaction between CD36 and TLR4 in oxLDL induced ECs, further activating the NF-κB signaling and enhancing endothelial inflammatory phenotype

**Fig. 7:**
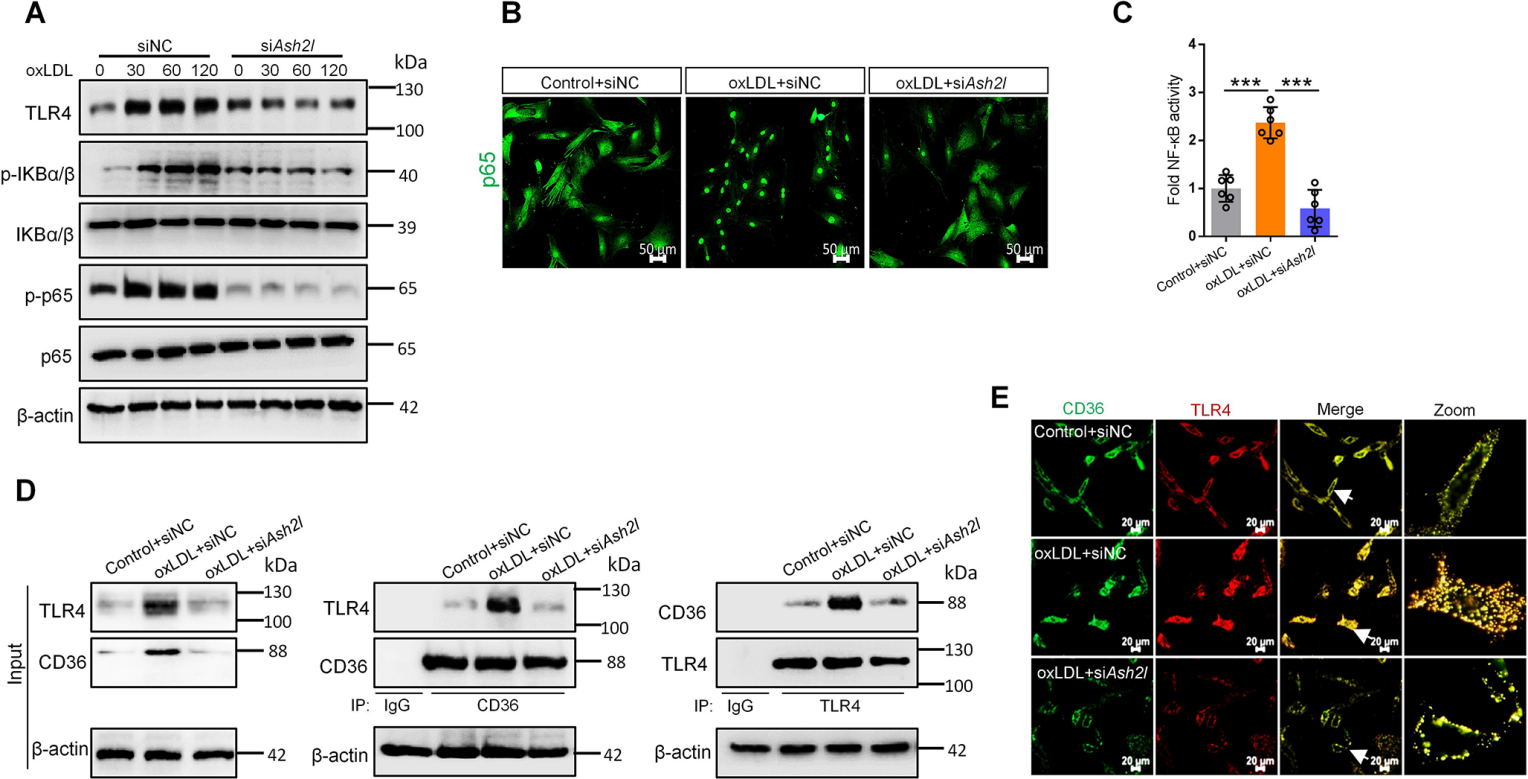
Ash2l promotes the interaction between CD36 and TLR4 in oxLDL induced ECs, further activating the NF-κB signaling and enhancing endothelial inflammatory phenotype. RAECs pre-transfected with siRNA *Ash2l* or siRNA control were incubated with 50 µg/mL oxLDL for indicated time. **A** Immunoblots analysis for TLR4 and NF-κB signal activation. β-actin was used as a loading control. **B** Immunofluorescence staining for p65; scale bars, 50 μm. **C** NF-κB activity was measured in oxLDL induced RAECs pre-transfected with siRNA *Ash2l* or siRNA control. Data were presented as the mean ± S.D, ^***^p < 0.001, *n* = 6. **D** Co-IP assay was performed to determine the combination of TLR4 and CD36. **E** Immunofluorescence staining for TLR4 (red) and the CD36 (green); Scale bars, 20 μm

The NF-κB signaling pathway regulated inflammatory responses and has been proved to be implicated in atherosclerosis. Incubation of ECs with oxLDL lead to significant phosphorylation of IKBα/β and p65 within 120 min without affecting their protein levels, and this process could be blocked by Ash2l silencing (Fig. 7A). Furthermore, knocking down of Ash2l reduced the nuclear translocation of p65 in oxLDL-treated ECs and abolished the enhanced activity of NF-κB caused by oxLDL (Fig. 7B and C). TLR4 was known to interact with CD36 to form a CD36-TLR dimer, which played a key role in the process of oxLDL-mediated NF-κB activation in macrophages [22]. Immunoblots analysis indicated that oxLDL indeed up-regulated TLR4 expression (Fig. 7A). To demonstrate the interaction between CD36 and TLR4 in ECs, immunoprecipitation assay for ECs lysates was performed using anti-CD36 or anti-TLR4. The results revealed that TLR4 co-precipitated with CD36 under basal conditions, and this effect was further enhanced with oxLDL stimulation, while knocking down of Ash2l significantly reduced the abundance of target proteins that interacted with bait antibodies in the precipitates (Fig. 7D). The co-localization of CD36 and TLR4 in ECs was further detected using confocal fluorescence microscopy. Minimal co-localization of CD36 and TLR4 was seen in control group. OxLDL treatment significantly improved the co-localization level of them and more distributed in the intracellular compartments, while knocking down of Ash2l relocated them to the cell membrane (Fig. 7E). In sum, these results suggested that oxLDL enhanced the interaction between CD36 and TLR4 in ECs and further triggered NF-κB signal transduction, which was related to the effect of Ash2l in promoting atherosclerosis.

## Discussion

Atherosclerosis has been considered as a chronic inflammatory disease. Endothelial dysfunction not only is certainly the initial factor or promoter of atherosclerosis, but also is critical in the transition from a stable to an unstable disease state. It is necessary to choose a promising avenue to prevent and treat endothelial dysfunction. Growing evidence indicates histone modification could regulate inflammatory response and vascular function. As a key histone modifying enzyme and an important member of the complex component WRAD, Ash2l can alter the chromatin architecture by mediating and enhancing the H3K4 methylation, and then influence the genome stability and lifespan. At present, Ash2l and its mediated H3K4 methylation are mainly reported in tumor-related research [23–25]. More and more evidence has shown Ash2l can regulate inflammatory response and vascular function, for example, our previous study showed Ash2l aggravated fibrosis and inflammation through HIPK2 transcriptional activation in diabetic nephropathy [26]; and aberrant histone methylation of IFN-γ associating H3K4me3 and H3K27me3 caused by over-binding of Ash2l and JMJD3 might be involved in vascular damage in Kawasaki disease in the acute phase [27]. In our present study, we identified that epigenetic factor Ash2l participated in the pathogenesis of atherosclerosis by aggravating vascular inflammation, promoting the uptake of oxidized lipids in ECs and weakening permeability barrier function. Mechanistically, Ash2l exacerbated endothelial inflammation through the transcriptional regulation of the scavenger receptors and NF-κB activation induced by enhancing proximity ligation signals between TLR4 and CD36. More importantly, Ash2l knockdown was associated with reduced scavenger receptors, which decreased the uptake of lipids by ECs and further improved the endothelial dysfunction. These findings revealed a causative role of Ash2l in the uptake of oxidized lipids and vascular inflammation in oxLDL induced ECs and suggested that Ash2l might be a potential therapeutic target in the early stage of atherosclerosis.

We firstly determine whether knocking down of Ash2l has an anti-atherosclerosis effect. Here we found that ECs-specific Ash2l knockdown could alleviate atherosclerotic lesions in both en-face and cross-sectional area in high-cholesterol diet *ApoE*^−/−^ mice, confirming Ash2l deficiency could prevent atherosclerosis. MMPs are essential to the instability of plaques. It has been reported inflammatory response could promote expression and release of MMPs by foam cells in the atherosclerotic plaque, thus ultimately reducing the stability of plaque and leading to plaque rupture [28–30]. Our result indicated that Ash2l deficiency increased collagen components and thickness of collagen cap, as well as decreased MMP2-positive areas, the MFI of MMP2 and the ratio of necrotic core area to lesion size in the plaques, suggesting that Ash2l played a key role in the formation and stability of late-stage atherosclerotic plaque, which might be related to Ash2l-mediated endothelial inflammatory response. We also detected multiple metabolic parameters (TC/TG/LDL-C/HDL-C) in mice fed with high-cholesterol diet, but did not find any significant difference in lipid profiles, which indicated that the pro-inflammatory and pro-atherosclerotic effects of Ash2l were not caused by abnormal lipid metabolism.

Scavenger receptors, as membrane-bounded carriers, are mainly expressed in macrophages and promote the uptake of oxLDL by macrophages to form foam cells [31]. As a direct contact of oxLDL, ECs can also promote the uptake and internalization of oxLDL through scavenger receptors on the surface of their cell membranes. It has been reported that scavenger receptor class B type 1 (SR-B1) in ECs mediates the uptake of LDL and transports it into subintima, thereby promoting atherosclerosis [32]. Endothelial overexpression of OLR-1 also increases plaque formation and promotes atherosclerosis in vivo by promoting oxLDL uptake and internalization [17, 33]. In our present study, we confirmed that the expression of scavenger receptors (OLR-1, CD36) and the uptake of oxLDL were increased upon oxLDL treated, which aggravated the production of inflammatory mediators by ECs. We also detected high expression of Ash2l in oxLDL induced ECs, while Ash2l knockdown significantly reduced the expression of scavenger receptors and the uptake of oxidized lipid, which might be an effective way to counteract atherosclerosis. Further, we proved that Ash2l bound to the promoter region of PPARγ and promoted its transcription by enhancing H3K4me3, which increased the expression of CD36 and OLR-1, consistent with the evidence that PPARγ could promote the expression of scavenger receptors and further increase the uptake and transport of free fatty acid [34, 35]. Notably, unlike the beneficial insulin sensitizing function in adipose tissue, the role of PPARγ in cardiovascular disease remains controversial [36]. Although multiple studies reported that PPARγ competitively inhibited the inflammatory signal pathway including NF-κB, JAK-STAT, NFTA (nuclear factor of activated T cell), and AP-1 (Activator protein-1), thereby alleviating inflammatory response and pathological damage of atherosclerosis [37–40], others suggested the activation of PPARγ exerted pro-inflammatory and pro-atherosclerotic effects [36]. Clinical trial data for type 2 diabetes showed that PPARγ agonist rosiglitazone could increase the incidence of adverse cardiovascular events [41]. In brief, our results showed Ash2l modulated PPARγ transcription activation through increasing promoter enrichment, which led to the upregulation of the direct PPARγ target genes in ECs, especially of OLR-1 and CD36, correspondingly increased the uptake and accumulation of oxidized lipids and aggravated atherosclerosis.

As the endogenous ligands, oxLDL was also reported to drive TLRs signal and resulted in the formation of CD36-TLR dimer to activate its downstream inflammatory signals, such NF-κB [42–45]. Our data corroborated above viewpoint that CD36 receptor accumulated on cell membrane and recruited TLR4 to enhance the proximity ligation signals between TLR4 and CD36, resulting in NF-κB activation and translocation. Loss of Ash2l abundance at the promoter of PPARγ inhibited CD36 transcription, which significantly weakened the proximity ligation signals between TLR4 and CD36, thus reducing NF-κB signal activation and contributing to the protection against endothelial injury and atherosclerosis.

In conclusion, we reported that Ash2l was a new regulator of endothelial injury and atherosclerosis, which was supported by two evidence chains: (1) Ash2l-PPARγ-SRs signaling axis drived oxidation lipid uptake by ECs; (2) CD36/TLR4-NF-κB signal axis activated ECs to produce a large number of inflammatory mediators, suggesting Ash2l might be an attractive therapeutic target for anti-atherosclerosis.

## Limitations

This study has potential limitations. Only selecting male mice for in vivo experiment may raise doubts about experimental design. In fact, sex-specific and hormone-related differences in atherosclerosis have been reported more than a decade ago [46]. Men develop atherosclerosis at younger ages and have higher mortality rates from atherosclerosis coronary artery disease than women [47, 48]. In addition to common risk factors such as smoking, hormones are thought to be an important reason for this difference. Since oestrogen exerts anti-atherosclerotic effects, atherosclerosis mainly occurs in postmenopausal women [48]. Given these variables (uncertain hormone changes et al.), this experiment only involved male mice to ensure consistency of experimental results. Also, the exact biological reasons behind the hormones or sex discrepancy deserve the future study.

### Electronic supplementary material

Below is the link to the electronic supplementary material.

Supplementary Material 1

## Data Availability

All data generated or analyzed during this study are included in this published article and its supplementary information files.

## Abbreviations

AAV-shRNA: Adeno-associated virus-endothelial specific system carrying
Ash2l: shRNA against Ash2l
AP-1: Activator protein-1
Ash2l: Absent, small, or homeotic-Like 2
ChIP-qPCR: Chromatin immunoprecipitation qPCR
ECs: endothelial cells
eNOS: endothelial nitric-oxide synthase
H3K4: histone H3 lysine 4
HDL-C: high-density lipoprotein cholesterol
ICAM: intercellular adhesion molecule
IHC: immunohistochemical
LDL-C: low-density lipoprotein cholesterol
LV-*Ash2l*: lentivirus-mediated *Ash2l*
MMP: matrix metallopeptidase
NFTA: nuclear factor of activated T cell
NF-κB: nuclear factor-kappa B
OLR-1: Lectin-like oxidized LDL receptor-1
OxLDL: Oxidized-low-density lipoprotein
PPARγ: peroxisome proliferator-activated receptor-γ.
RAECs: rat artery endothelial cells
SDS-PAGE: sodium dodecyl sulfate-polyacrylamide gel electrophoresis
siRNA: Small interfering RNA
SR-A: scavenger receptor class A
SR-B1: scavenger receptor class B type 1
TC: total cholesterol
TG: triglyceride
TLR4: toll-like receptor 4
VCAM: vascular adhesion molecule
