# Supplementary material for: Functional role of Ash2l in oxLDL induced endothelial dysfunction and atherosclerosis
Source: Cell Mol Life Sci. 2024 Jan 27;81(1):62. doi: 10.1007/s00018-024-05130-5 (PMC10821849; doi:10.1007/s00018-024-05130-5)
Supplement: Supplementary file 1 — Supplementary Material 1 [file 18_2024_5130_MOESM1_ESM.docx]

**Supplementary materials and methods**

**Functional role of Ash2l in oxLDL induced endothelial dysfunction and atherosclerosis**

Zhenghua Su, Jinghuan Wang, Chenxi Xiao, Wen Zhong, Jiayao Liu, Xinhua Liu^*^, YiZhun Zhu^*^

*Correspondence should be addressed to: [liuxinhua@fudan.edu.cn](mailto:liuxinhua@fudan.edu.cn) and [yzzhu@must.edu.mo](mailto:yzzhu@must.edu.mo)

**This file includes:**

Materials and Methods

Supplemental Figures S1-S6

Supplementary Tables S1-S2

Other Major Resources Tables

**Materials and Methods**

**Reagents and antibodies**

OxLDL was obtained from Yeasen Biotechnology (Shanghai, China). Cell culture reagents were obtained from HyClone (Logan, UT, USA) unless stated otherwise. Antibodies to Ash2l, PPARγ, Total H3, p65, p-p65, IκBα and p-IκBα were obtained from Cell Signaling Technology (Danvers, MA, USA). Antibodies to IL-6, IL-1β and Cox-2 were purchased from ABclonal (Wuhan, China). Antibodies to OLR-1, CD36, VCAM-1, iNOS, β-Actin, β-tubulin and GAPDH were purchased from Proteintech (Rosemont, IL, USA). Antibodies to CD36 was obtained from ThermoFisher Scientific (Waltham, MA, USA). Antibodies to MSR and sodium potassium ATPase were obtained from Bioss (Beijing, China). Antibodies to MMP2 and eNOS were bought from Servicebio Technology (Wuhan, China). Antibodies to H3K4me3, and Claudin-5 were purchased from Abcam (Cambridge, MA, USA). Antibodies to ICAM-1, TLR4 and Cox-2 were obtained from Santa Cruz Biotechnology and used for immunofluorescence. Antibodies to CD31 was purchased from R&D Systems.

**Construction of AAV-shRNA *Ash2l* (adeno-associated virus-endothelial specific system carrying shRNA against Ash2l) vector**

Target insertion seq (gcCGGACACCTACAAAGATAA) and control insertion seq (TTCTCCGAACGTGTCACGT) were produced and used to prepare DNA oligo. The GV726 AAV-shRNA vector (pAAV-ICAM2p-EGFP-MIR155(MCS)-SV40 PolyA) was obtained from Genechem co. ltd (Shanghai, China). Above prepared effective target or control oligonucleotide was inserted into pAAV-ICAM2p-EGFP-MIR155(MCS)-SV40 PolyA to construct AAV-shRNA *Ash2l* vector or AAV-shRNA *control* vector. ECs-specific ICAM2 promoter element in GV726 AAV-shRNA vector was for better delivery AAV-shRNA *Ash2l* to ECs in vivo.

**Mice and atherosclerosis model induction**

All experimental procedures in mice were approved by the Fudan University’s Institutional Animal Care and Usage Committee and complied with all relevant ethical regulations. Only males were selected in our study to avoid potential interference of hormones.

8-10 weeks old male *ApoE*^−/−^ mice were acclimated to the environment for two days and then fed a high-cholesterol diet (TP26300, Trophic Animal Feed High-Tech Co., Ltd, China) for 12 weeks. Meanwhile, male C57BL/6J mice with the same weight were fed with chow diet as control. For prevention, 1 × 10^12^ vg adeno-associated virus (AAV)-endothelial specific system carrying shRNA against *Ash2l* (AAV-shRNA *Ash2l*) or negative control (AAV-shRNA Ctrl) was injected through tail-vein on the first week of high-cholesterol diet. After 12 weeks, all mice were euthanized and perfused with physiological saline, after which the aortic arteries were harvested. 8-16 8-µm-thick sections were obtained from each sample's aortic sinus for the plaque analysis.

**Lipids analysis and lipoprotein profile measurement.**

Mice with different treatment were fasted for 12 h and blood samples were collected in blood collection tube pretreated with sodium EDTA. Serum was obtained from pooled plasma by centrifugation at 3500 rpm, 4°C for 15 min and then stored at -80°C. The concentrations of total cholesterol (TC), triglycerides (TG), low-density lipoprotein cholesterol (LDL-C) and high-density lipoprotein cholesterol (HDL-C) in blood serum were enzymatically measured with corresponding TC/TG/LDL-C/HDL-C Quantitation Kit (Nanjing Jiancheng Bioengineering Institute, China) as recommended by manufacturer’s instructions.

**Proteomic analysis of human samples**

Pristine (not yet stenosis or occlusion), stenosis, and occlusion arteries from patients with atherosclerosis (Pa/Sa/Oa) or atherosclerosis and diabetes (Pd/Sd/Od) were collected in accordance with the guidelines of the Sun Yat-sen Memorial Hospital and the Fifth Affiliated Hospital of Zunyi Medical University. Proteome analysis for artery samples was performed in novogene company (Beijing, China). Proteins related to inflammation and scavenger receptors were selected for differential expression analysis in a heat map format.

**Histological analysis of atherosclerotic lesion**

For cross-sectional analysis of aortic sinus, aortic roots were embedded in OTC to make 8-µm-thick serial cryosections. The morphology of aortic sinus was evaluated by staining with hematoxylin and eosin (H&E). Collagen contents in cryosections of aortic root were examined using Masson’s trichrome staining Kit (R20381, Yuanye Bio-Technology Co., Ltd, Shanghai, China) as recommended by manufacturer’s instructions.

Oil red O staining was performed to evaluate the lipid contents of aortic root. In brief, cross-sections of aortic root were stained with Oil red O working solution for 30 min followed by removing nonspecific staining with 60% isopropanol for about 5 seconds. For en-face lipid contents analysis, the entire aortas were isolated and stained with Oil Red O working solution followed the protocol described above. All images were captured and processed using identical settings in the fluorescence microscope (Axio Scope.A1, Carl Zeiss Imaging Systems) and quantified with Image J Software.

**Rat primary endothelial cells isolation and induction**

80-100 g Male Sprague-Dawley rats were euthanized and perfused with physiological saline, aortic arteries were then harvested and sliced into several vascular rings. After incubated with DMEM containing collagenase I (2.0 g/L) for 1 h at 37°C, the vascular rings were adhered to the collagen coated culture bottle with the intima downward and cultured in ECM medium containing penicillin-streptomycin (15140122, Gibco, ThermoFisher Scientific, Waltham, MA, USA) and endothelial growth factor at 37°C in a 5% CO_2_ incubator. 24 h latter, cells migrated out of the vascular rings and were cultured in DMEM medium (Gibco, ThermoFisher Scientific, Waltham, MA, USA) supplemented with 10% fetal bovine serum (FBS, 10099141, Gibco, ThermoFisher Scientific, Waltham, MA, USA) and 1% penicillin-streptomycin at 37°C in a humidified incubator with 5% CO_2_. Rat primary endothelial cells (RAECs) were identified by immunofluorescence staining using CD31 antibody and the cells passages of 3-6 were used for the future experiments. 50 μg/mL oxLDL was used to induce RAECs inflammation and dysfunction in vitro.

**Small interfering RNA (siRNA) transfection**

Rat *PPARγ*, Rat *Ash2l*, Homo *Ash2l* siRNA and control siRNA were produced by GenePharma (Shanghai, China). Lipofectamine RNA iMAX, *Ash2l* siRNA (at 25 to 50 nM), and Opti-MEM was mixed and incubated at room temperature for 5 min. Cells at 30% to 50% confluence were incubated with the prepared siRNA-lipofectamine RNA iMAX complexes for 8-24 h and the medium was then replaced by fresh serum DMEM medium. The Ash2l knockdown efficiency verified by western blot 72 h post-transfection.

**Lentivirus generation and infection**

Rat *Ash2l* cDNA expression lentiviral vectors were obtained from Miaolingbio (Wuhan, China). Ash2l expression plasmid, recombinant plasmid and packaging vectors psPAX2 and PMD2.G were co-transfected into HEK293 cells. We collected culture media 48 h and 72 h after transfection respectively and then removed the cell fragments with a 0.45 μm filter membrane to obtain the virus suspension, which was further added into the culture medium of RAECs for 24 h and the medium was then replaced with fresh serum DMEM medium. The Ash2l expression was verified by western blot 72 h post-transfection.

**Immunohistochemical (IHC)**

Protein expression in cryosections of aortic root was examined using mouse and rabbit specific HRP/DAB detection immunohistochemical staining Kit (ab64264, Abcam) as recommended by manufacturer’s instructions. In brief, endogenous peroxidase activity of cryosections was quenched by Hydrogen Peroxide Block, and nonspecific sites were blocked up by incubating with Protein Block at room temperature. Next, cryosections were incubated overnight with appropriate amounts of primary antibodies at 4°C followed by combination with HRP-conjugated secondary antibodies for 1 h at 37°C and then stained with DAB working solution for another 10 min. After nuclei were stained with hematoxylin for 1 min, the protein expression was visualized using a fluorescence microscope (Axio Scope.A1, Carl Zeiss Imaging Systems).

**Immunofluorescence**

RAECs were cultured on coverslips placed in cell cultured plate. Following different treatments, cells were fixed in 4% paraformaldehyde for 15 min, permeabilized with 0.25% Triton X-100 in PBS for 10 min, and blocked with 10% goat serum in PBS for 30 min at room temperature. The slides were incubated overnight with appropriate amounts of primary antibodies or IgG at 4°C and then binding to the corresponding secondary antibodies for another 2 h at 37°C. DAPI was used to display and locate nuclei. For tissue sample, cryosections of aortic root were fixed in acetone, and processed for antibodies following the protocol described above. Images were captured using a fluorescence microscope (Axio Scope.A1, Carl Zeiss Imaging Systems).

**Western blotting**

Cultured cells and tissue proteins were lysed with RIPA buffer (Pierce, Rockford, IL, USA) containing a cocktail of protease (K1019, APExBIO) and phosphatase (K1015, APExBIO) inhibitors and 5% β-mercaptoethanol (60-24-2, Aladdin). Protein concentration was measured with Pierce^TM^ BCA Protein Assay Kit (23227, ThermoFisher Scientific). Whole protein samples were separated via sodium dodecyl sulfate-polyacrylamide gel electrophoresis (SDS-PAGE) and then transferred to nitrocellulose membranes. Next, membranes were incubated in blocking buffer (5%, w/v, dried skimmed milk in TBST) for 2 h and then incubated with appropriate amounts of primary antibodies diluted in blocking buffer overnight at 4°C. HRP-conjugated secondary antibodies (Jackson ImmunoResearch) were added to the immune membranes for 2 h at room temperature and western blot images were acquired by ChemiDoc+ (Bio-RAD, Hercules, CA) and quantified using Image J Software.

**Quantitative Real-Time PCR**

Total RNA was extracted with Trizol reagent (9109, Takara) and reverse-transcribed into cDNA and amplified using a PrimeScriptTM1st Strand cDNA Synthesis Kit (Takara, China) as recommended by the manufacturer’s directions. Quantitative real-time PCR analysis was performed using iCycler iQ system (Bio-Rad, Hercules, CA). Threshold cycle (Ct) values of β-actin were subtracted from Ct values of the genes of interest (^Δ^Ct). The primers sequences for PCR were listed in Supplemental **Table S1**.

**Plasma membrane separation and detection by immunoblot analysis**

RAECs were incubated with or without oxLDL (50 µg/mL) for 15 min or 24 h after transfected with *Ash2l* siRNA or negative control, the plasma membrane proteins were then isolated using the Mem-PER™ Plus Plasma Membrane Protein Isolation Kit as recommended by manufacturer’s instructions (ThermoFisher Scientific), which offers rapid isolation of plasma membrane proteins from cultured cells without contamination with organelles and other membranous fractions. The fraction containing plasma membrane proteins was analyzed by immunoblot.

**Dil-oxLDL uptake assay**

To assess the uptake of oxLDL, RAECs were incubated with Dil-labeled oxLDL (50 μg/mL, Yeasen Biotechnology, Shanghai, China) for 4 h at 37°C, cells were then washed three times with cold PBS and fixed with 4% paraformaldehyde. The nucleus was stained with DAPI to locate the cell. Fluorescence intensity was assessed under a fluorescence microscope (Axio Scope.A1, Carl Zeiss Imaging Systems) and quantified with Image J Software.

**Chromatin immunoprecipitation PCR (ChIP-PCR)**

ChIP followed by qPCR was used to examine Specific protein-DNA interactions. Briefly, RAECs treated with or without 50 μg/mL oxLDL were crosslinked with 1% formaldehyde for 10 min at room temperature and fragmented to lengths between 200 and 500 base pairs by sonicator, respectively. Then pre-cleared fragments were incubated overnight with Ash2l, H3K4me3 and PPARγ antibody or IgG control. Immunoprecipitated protein-DNA complexes were purified, followed by captured with protein-G beads (10003D, Invitrogen). Primers for ChIP assay were designed within a range of 0-500 bp before the promoter regions of PPARγ, CD36 and OLR-1 gene, and PCR analysis was performed to obtain products of approximately 200 bp in size. The results were presented as visualization and quantification of the gel for the PCR products. Primer sequences for ChIP assay were listed in Supplementary **Table S2.**

**Co-immunoprecipitation**

Following different treatments, RAECs were lysed with the RIPA buffer containing proteinase inhibitor and phosphatase inhibitor and centrifuged at 13,000 g for 15 min to remove the debris. Cell lysates were quantified using Pierce^TM^ BCA Protein Assay Kit (ThermoFisher Scientific) and then incubated with appropriate amounts of antibodies (IgG antibody as control) at 4°C for 1 h based on instructions. After incubation with 20 μL Protein A/G PLUS-Agarose (sc-2003, Santa Cruz Biotechnology) at 4°C for overnight on a rocker platform, immunoprecipitates were collected by centrifugation at 2500 rpm for 5 min at 4°C, washed four times with RIPA buffer and boiled in sample loading buffer for SDS-PAGE and autoradiography.

**Dural luciferase reporter assay**

The pGL6-TA firefly luciferase plasmid containing multiple NF-κB binding sites were constructed by Beyotime Biotechnology (D2207, pNF-κB-TA-luc). PPARγ luciferase reporter plasmid (PPARγ-luc) was obtained from Yeasen Biotechnology (Shanghai, China). The pRL-TK plasmid carrying the Renilla luciferase reporter gene (D2760, Beyotime Biotechnology) was used as internal control for the detection of firefly luciferase reporter gene to eliminate the error caused by the different transfection efficiency of the plasmid. The pNF-κB-TA-luc or PPARγ-luc was transfected into RAEC, together with pRLTK plasmid containing the Renilla luciferase reporter gene. Six hours after transfection, cells were treated with oxLDL (50 µg/mL) for 24 h. Firefly and Renilla luciferase activities were then measured with the Dual Luciferase Reporter Gene Assay Kit (RG027, Beyotime Biotechnology).


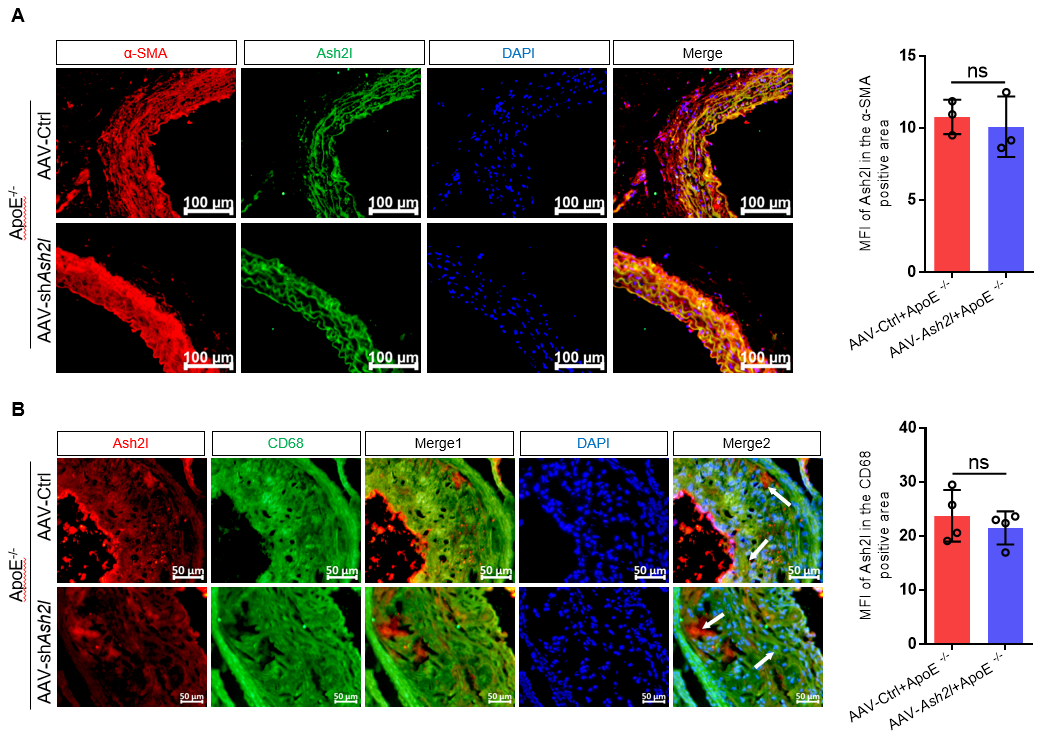


**Figure S1. ECs-Specific Ash2l knockdown does not affect the expression of Ash2l in vascular smooth muscle cells (VSMCs) and monocytes/macrophages.** **(A-B)** Immunofluorescence staining for α-SMA (red)/Ash2l (green) in aortic wall **(A)** or CD68 (green)/Ash2l (red) in cross-sections of the aortic roots **(B)** was compared between AAV-shRNA *Ash2l* and AAV-*Ctrl* treated *ApoE*^−/−^ mice; scale bars, 100 μm or 50 μm. Data were presented as the mean ± S.D, *P* values were calculated by or two-tailed Student’s t-test, ns meant no significance, n=3 or n=4.


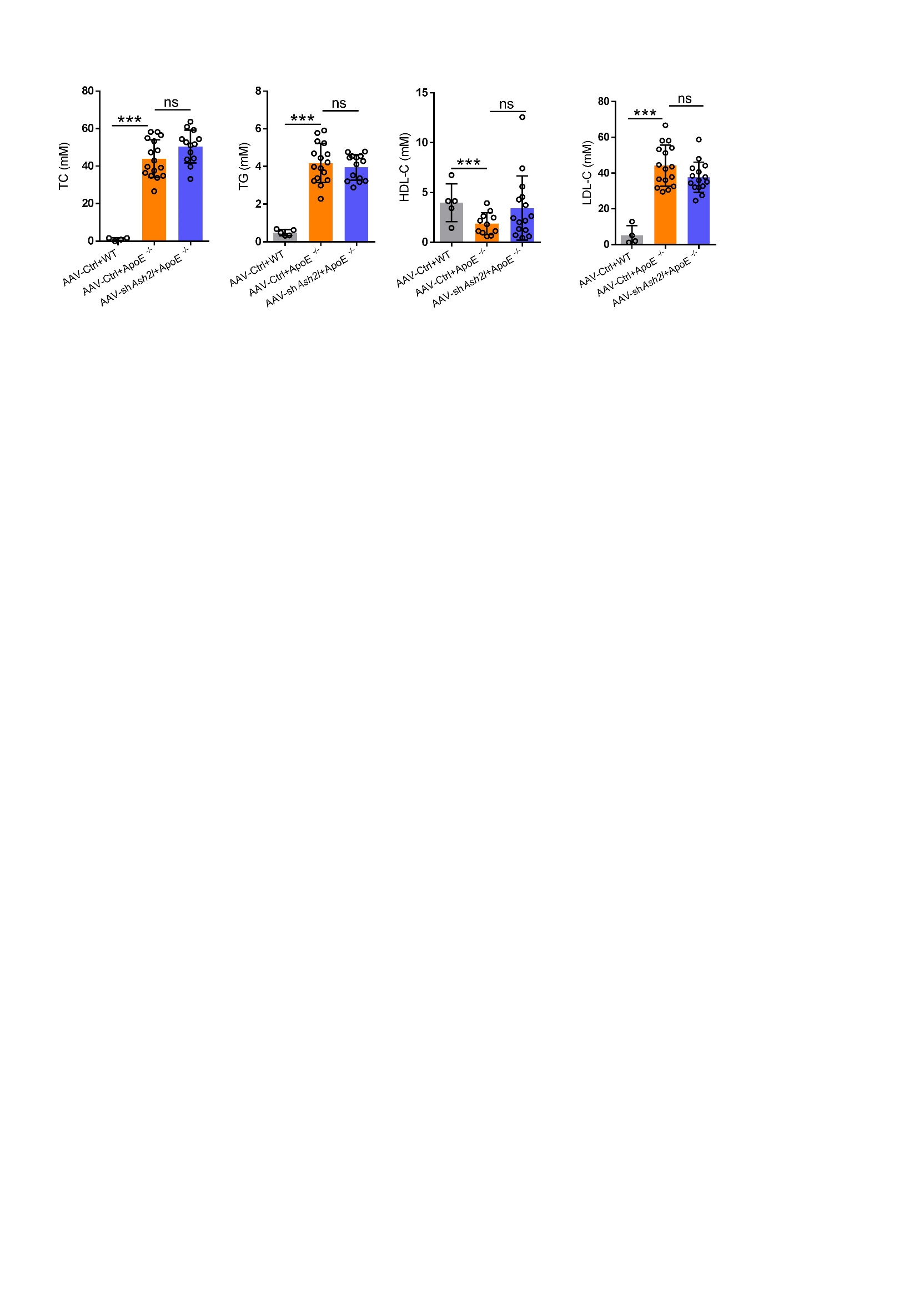


**Figure S2. ECs-Specific Ash2l knockdown does not affect multiple metabolic parameters in serum.** TC, TG, LDL-C and HDL-C levels were detected in serum from mice fed with chow or high-cholesterol diet 12 weeks. Data were presented as the mean ± SD, *P* values were calculated by One-way ANOVA test, ^***^*p* < 0.001, ns meant no significance, n=4-16/group.


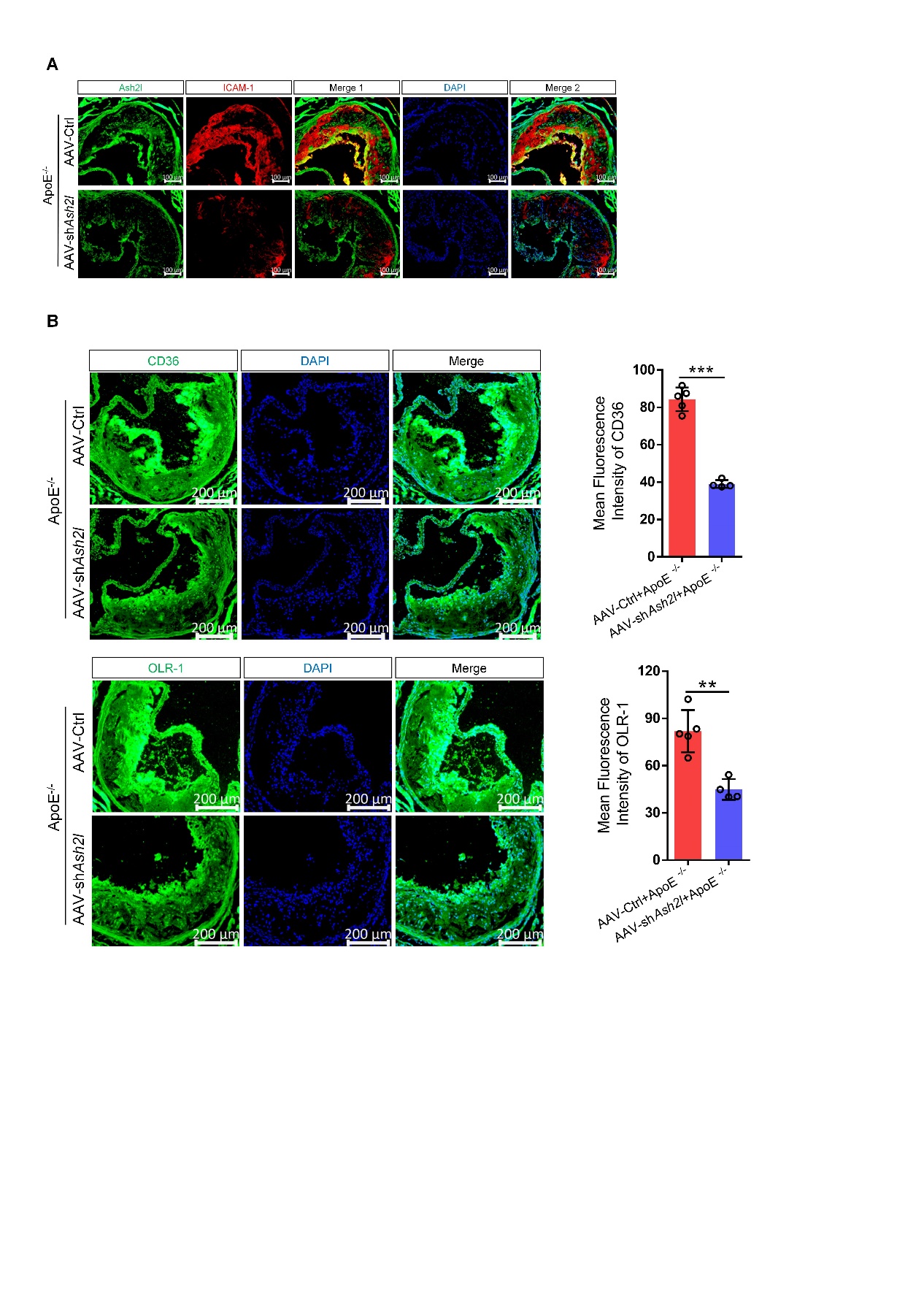


**Figure S3. ECs-Specific Ash2l knockdown inhibits inflammatory mediators and scavenger receptor expression in vivo.** **(A)** Immunofluorescence staining for Ash2l (green) and ICAM-1 (red) in cross-sections of the aortic roots from AAV-shRNA *Ash2l* and AAV-*Ctrl* treated *ApoE*^−/−^ mice; scale bars, 100 μm. **(B)** Immunofluorescence staining for scavenger receptors (CD36, OLR-1) in cross-sections of the aortic roots from AAV-shRNA *Ash2l* or AAV-*Ctrl* treated *ApoE*^−/−^ mice; scale bars, 200 μm. Right panel: quantification for the mean fluorescent intensity (MFI) of CD36 or OLR-1 in each group, n=4-5. Data were presented as the mean ± S.D, *P* values were calculated by two-tailed Student’s t-test, ^**^*p* < 0.01, ^***^*p* < 0.001.


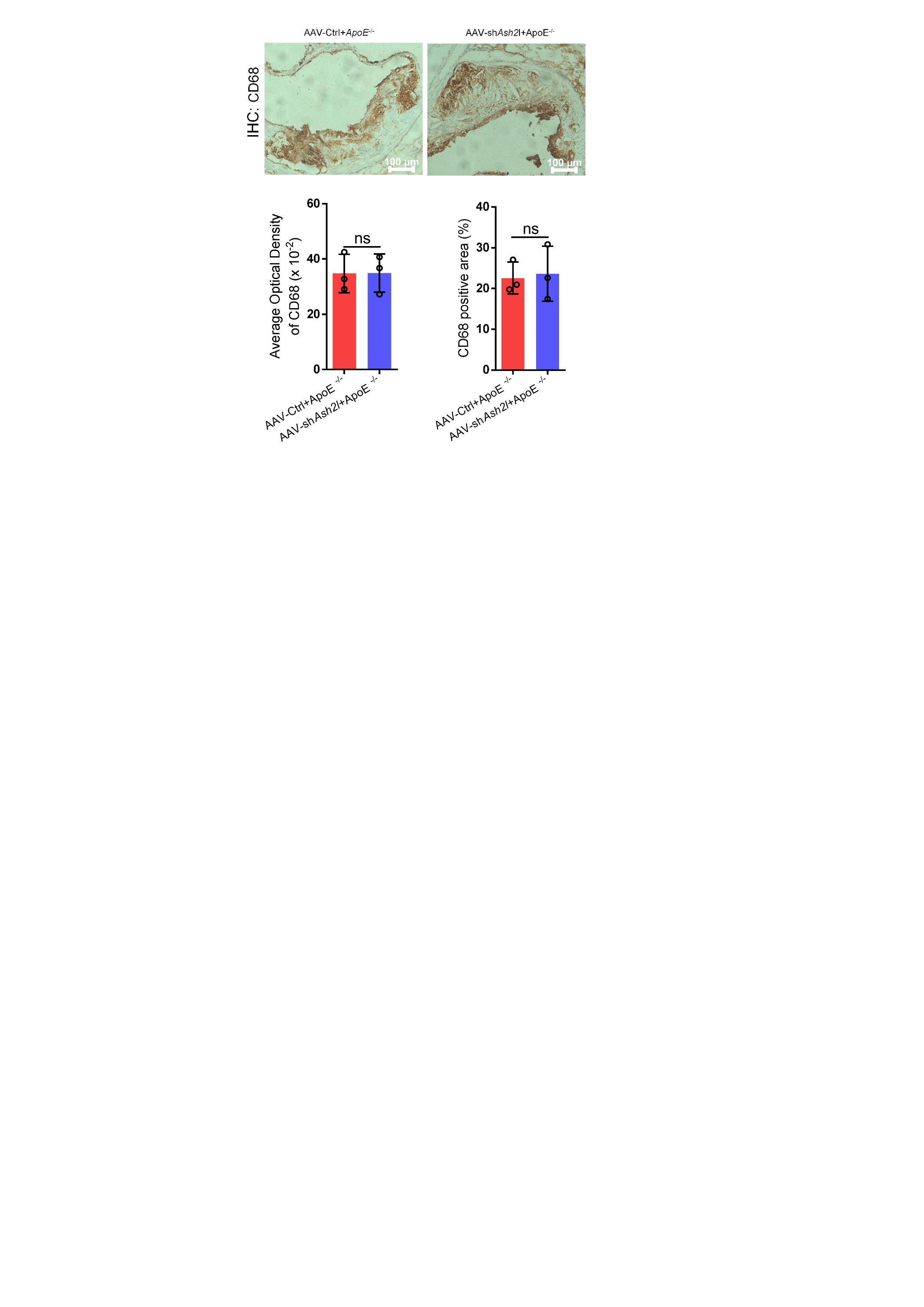


**Figure S4. ECs-Specific Ash2l knockdown has little effect on macrophage retention.** Immunohistochemical staining for CD68 in atherosclerotic lesions was compared between AAV-shRNA *Ash2l* and AAV-*Ctrl* treated *ApoE*^−/−^ mice; scale bars, 100 μm. The bottom panel indicated the quantification for positive area or average optical density (AOD) of CD68. Data were presented as the mean ± SD, *P* values were calculated by One-way ANOVA test, ns meant no significance, each acquired from three independent experiments.


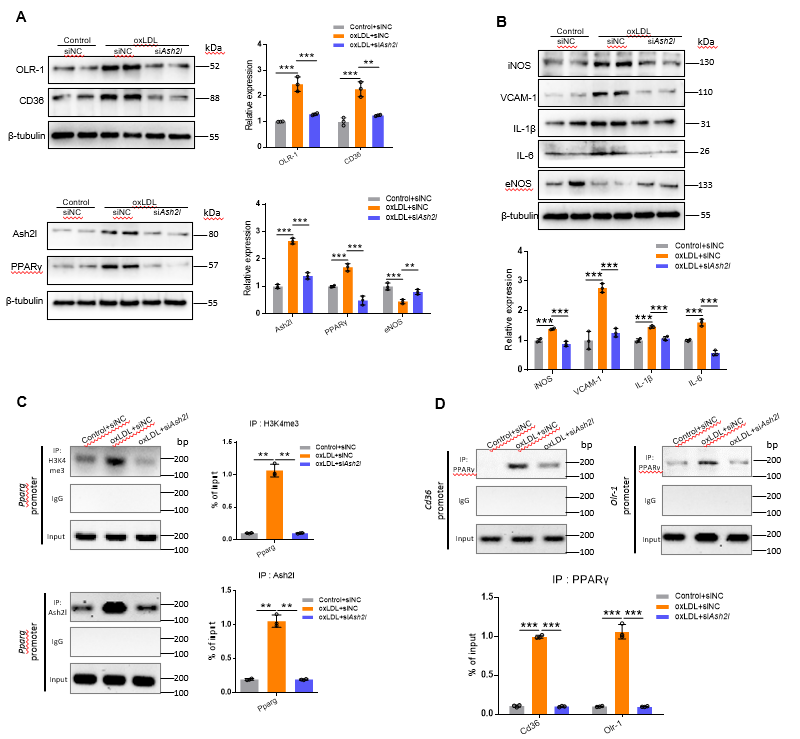


**Figure S5. The major outcomes were reproduced in Human umbilical vein endothelial cells (HUVECs).** HUVECs pre-transfected with siRNA *Ash2l* or siRNA control were incubated with 50 µg/mL ox-LDL for indicated time. **(A-B)** Immunoblots analysis was performed to determine protein expression of Ash2l, PPARγ, scavenger receptors (CD36, OLR-1) **(A)** and inflammatory mediators (iNOS, VCAM-1, IL-6, IL-1β, eNOS) **(B)**, and quantification of these protein expression was shown in the figure right **(A)** or figure bottom **(B)**. (**C**) ChIP-qPCR assays were performed to determine the level of H3K4me3 and Ash2l at the *Pparg* promoter region, the visualization and quantification of the gel for the PCR products were shown in figure. (**D**) ChIP-qPCR assays were performed to determine the combination of PPARγ and the promoter region of Scavenger receptors (*Cd36, Olr-1*) in ox-LDL induced HUVECs, the visualization and quantification of the gel for the PCR products were shown in figure. All data were presented as the mean ± S.D, P values were calculated by One-way ANOVA test, ^**^*p* < 0.01, ^***^*p* < 0.001, each acquired from three independent experiments.


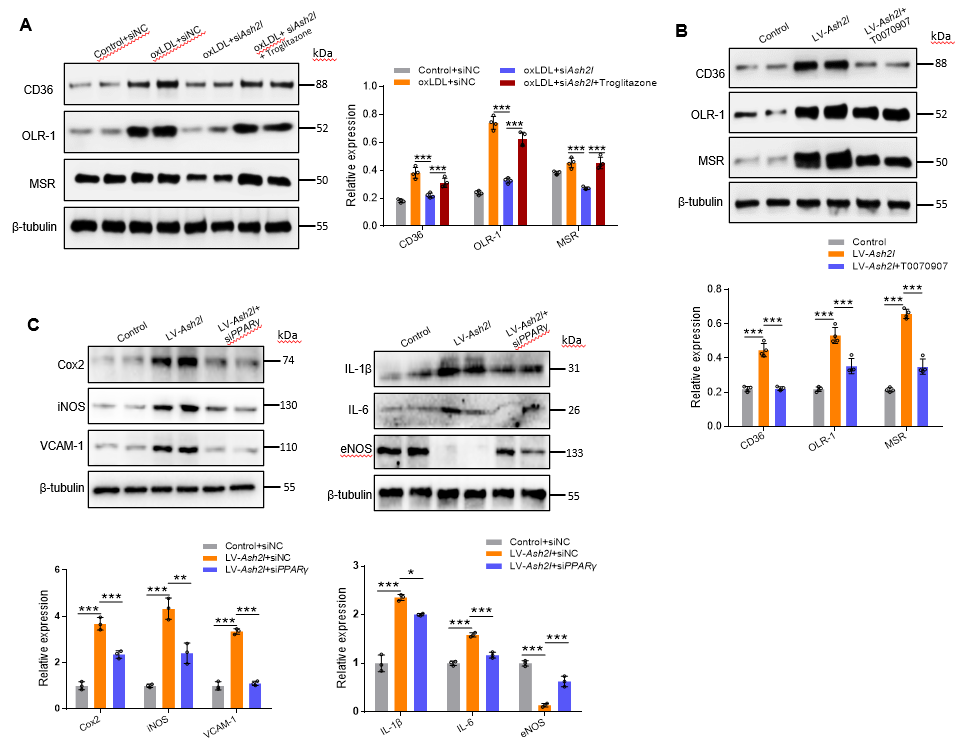


**Figure S6. Ash2l-mediated the expression of scavenger receptors is largely PPARγ-dependent. (A)** The protein expression and quantification for scavenger receptors (CD36, OLR-1, MSR) in oxLDL-treated RAECs pre-transfected with siRNA *Ash2l* and followed incubated with Troglitazone. **(B)** PPARγ antagonist T0070907 counteracted the high expression of scavenger receptors (CD36, OLR-1, MSR) triggered by Ash2l overexpression, quantification for scavenger receptors expression was shown in figure bottom. **(C)** RAECs were pre-infected with lentivirus-mediated *Ash2l* cDNA and then transfected with siRNA *PPARγ*, immunoblots assay was performed to determine protein expression inflammatory mediators (Cox2, iNOS, VCAM-1, IL-6, IL-1β, eNOS)**,** and quantification of these protein expression was shown in the figure bottom. Data were presented as the mean ± SD, *P* values were calculated by One-way ANOVA test, ^*^*p* < 0.05, ^**^*p* < 0.01, ^***^*p* < 0.001, each acquired from three independent experiments.

**Supplementary Table S1**

The primers sequences for RT-qPCR.

| Name | Sequence |
| --- | --- |
| Rat_*Ash2l*_F | AGTGGGAACCTAAATGGGGG |
| Rat_*Ash2l*_R | AGGAATAGGTTTGCCTGCCC |
| Mus_*Ash2l*_F | AGAAGGGAGGTCAACTGGAG |
| Mus_*Ash2l*_R | CGCCTGGGTATCCATCACTT |
| Rat_ *Pparg*_F | TCCCGTTCACAAGAGCTGAC |
| Rat_ *Pparg*_R | ATAATAAGGCGGGGACGCAG |
| Rat_*Cd36*_F | TCTCAATCTGGCTGTGGCAG |
| Rat_*Cd36*_R | GGCTGTTGAGCACACCTTGA |
| Rat_*Olr1*_F | TCAGGGCTAGCAATGGGAATC |
| Rat_*Olr1*_R | GTTCACCAAGGCAGCTTGTC |

**Supplementary Table S2**

The primers position and sequences for ChIP-PCR.

**
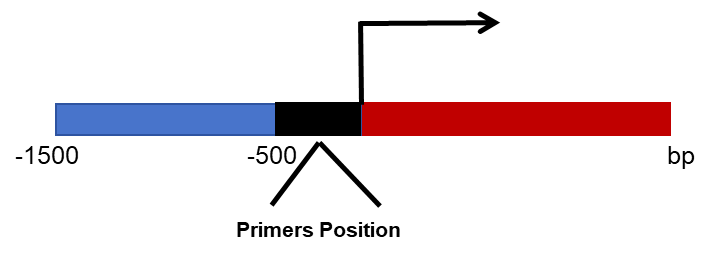
**

| Name | Sequence |
| --- | --- |
| Rat_*Cd36*_F | GCTGGATGCCAGGCTTACTT |
| Rat_*Cd36*_R | GCCCCAAGTCAAATCACTGC |
| Rat_*Olr1*_F | TCAGGGCTAGCAATGGGAATC |
| Rat_*Olr1*_R | GTTCACCAAGGCAGCTTGTC |
| Rat_ *Pparg*_F | AGGTGTTCTCCTTCATATTGTTCT |
| Rat_ *Pparg*_R | TCCCACGTTCCTCAGACAGA |
| Human_*Cd36*_F | TGACTCTGTGGCTGCTCTCT |
| Human _*Cd36*_R | ATGCAGCTACCTTTGAGCCAC |
| Human _*Olr1*_F | GGTGCTGGGCATGCAATTATC |
| Human _*Olr1*_R | TGGATGAAGTCCTGAACAATTTGC |
| Human _ *Pparg*_F | TGACTGAACCCCCTGTTGTG |
| Human _ *Pparg*_R | GGGCTTGTAGCAGGTTGTCT |

**Other Major Resources Table**

**Genetically Modified Animals**

|  | **Species** | **Vendor or Source** | **Background Strain** | **Other Information** |
| --- | --- | --- | --- | --- |
| **Parent - Male** | Mus musculus | SHANGHAI SLAC LABORATORY ANIMAL CO. LTD | C57BL/6J | *Apoe* KO |
| **Parent - Female** | Mus musculus | SHANGHAI SLAC LABORATORY ANIMAL CO. LTD | C57BL/6J | *Apoe* KO |

**Antibodies**

| **Target antigen** | **Vendor or Source** | **Catalog #** | **Working concentration** | **Applicati on** |
| --- | --- | --- | --- | --- |
| Ash2l | Cell Signaling Technology | #5019S | 1:2000 | WB |
| PPARγ | Cell Signaling Technology | #2435T | 1:1000 | WB |
| Histone H3 | Cell Signaling Technology | #4499 | 1:2000 | WB |
| p65 | Cell Signaling Technology | #3034 | 1:1000 | WB |
| p-p65 | Cell Signaling Technology | #3031S | 1:1000 | WB |
| IκBα | Cell Signaling Technology | #4814T | 1:1000 | WB |
| p-IκBα | Cell Signaling Technology | #2859T | 1:1000 | WB |
| IL-6 | ABclonal | A0286 | 1:1000 | WB |
| IL-1β | ABclonal | A1112 | 1:1000 | WB |
| Cox-2 | ABclonal | A1253 | 1:1000 | WB |
| OLR-1 | Proteintech | 11837-1-AP | 1:1000 | WB |
| CD36 | Proteintech | 18836-1-AP | 1:1000 | WB |
| VCAM-1 | Proteintech | 11444-1-AP | 1:1000 | WB |
| iNOS | Proteintech | 18985-1-AP | 1:1000 | WB |
| β-Actin | Proteintech | 66009-1-Ig | 0.1 μg/ml | WB |
| β-tubulin | Proteintech | 66240-1-Ig | 0.1 μg/ml | WB |
| GAPDH | Proteintech | 60004-1-Ig | 0.1 μg/ml | WB |
| MSR | Bioss | bs24362R | 1 μg/ml | WB |
| sodium potassium ATPase | Bioss | bsm-52485R | 1:1000 | WB |
| eNOS | Servicebio Technology | GB11086 | 1:1000 | WB |
| H3K4me3 | Abcam | ab8580 | 1 μg/ml | WB |
| Claudin-5 | Abcam | ab131259 | 1:1000 | WB |
| TLR4 | Santa Cruz Biotechnology | sc-293072 | 1:500 | WB |
| ICAM-1 | Santa Cruz Biotechnology | sc-107 | 1:200 | IF |
| TLR4 | Santa Cruz Biotechnology | sc-293072 | 1:200 | IF |
| Cox-2 | Santa Cruz Biotechnology | sc-514489 | 1:200 | IF |
| CD31 | BD Bioscience | 550274 | 1:250 | IF |
| PPARγ | Cell Signaling Technology | #2435T | 1:200 | IF |
| p-p65 | Cell Signaling Technology | #3031S | 1:200 | IF |
| CD36 | ThermoFisher Scientific | #PA1-16813 | 1:200 | IF |
| MMP2 | Servicebio Technology | GB11130 | 1:500 | IF |
| Ash2l | Cell Signaling Technology | #5019S | 1:500 | IF |
| Ash2l | Santa Cruz Biotechnology | sc-81184 | 1:300 | IF |
| CD68 | Servicebio Technology | GB11067 | 1:300 | IF |
| α-SMA | Servicebio Technology | GB13044 | 1:400 | IF |
| Ash2l | Cell Signaling Technology | #5019S | 1:100 | ChIP |
| PPARγ | Cell Signaling Technology | #2435T | 1:100 | ChIP |
| Histone H3 | Cell Signaling Technology | #4499 | 2 µg /25 µg chromatin | ChIP |

**DNA/cDNA Clones**

| **Clone Name** | **Sequence** | **Source / Repository** |
| --- | --- | --- |
| Rat *Ash2l* siRNA | 5’-GGAAUAGACACGUCGUCAUTT-3’ | GenePharma |
| Rat *PPARγ* siRNA | 5’-GAAUACCAAAGUGCGAUCATT-3’ | GenePharma |
| Rat *control* siRNA | 5’-UUCUCCGAACGUGUCACGUTT-3’ | GenePharma |
| Homo *Ash2l* siRNA | 5’-GCUGCACGGUUUCCAUUAATT-3’ | GenePharma |
| Homo *control* siRNA | 5’-UUCUCCGAACGUGUCACGUTT-3’ | GenePharma |

**Cultured Cells**

| **Name** | **Vendor or Source** | **Sex (F, M, or unknown)** |
| --- | --- | --- |
| Rat artery endothelial cells (RAECs) | Primary Culture | M |
| Human umbilical vein endothelial cells (HUVECs) | Primary Culture | M |

**Other**

| **Description** | **Source / Repository** |
| --- | --- |
| oxidized LDL (oxLDL) | Yeasen Biotechnology |
